# Medicinal plant *Miconia albicans* synergizes with ampicillin and ciprofloxacin against multi-drug resistant *Acinetobacter baumannii* and *Staphylococcus aureus*

**DOI:** 10.1186/s12906-023-04147-w

**Published:** 2023-10-23

**Authors:** Genilson Silva de Jesus, Danielle Silva Trentin, Thayná Fernandes Barros, Alda Maria Teixeira Ferreira, Bruna Castro de Barros, Patrícia de Oliveira Figueiredo, Fernanda Rodrigues Garcez, Érica Luiz dos Santos, Ana Camila Micheletti, Nidia Cristiane Yoshida

**Affiliations:** 1 Laboratório de Produtos Naturais Bioativos-PRONABio, Instituto de Química, Universidade Federal de Mato Grosso Do Sul, Campo Grande, Brazil; 2 Laboratório de Bacteriologia & Modelos Experimentais Alternativos (BACMEA), Departamento de Ciências Básicas da Saúde, Universidade Federal de Ciências da Saúde de Porto Alegre, Porto Alegre, Brazil; 3 Instituto de Biociências, Universidade Federal de Mato Grosso Do Sul, Campo Grande, Brazil

**Keywords:** MRSA, Antibiotic resistance, Antibiofilm, Synergism, Secondary metabolite, Bioactive natural products

## Abstract

**Background:**

Given the rising occurrence of antibiotic resistance due to the existence and ongoing development of resistant bacteria and phenotypes, the identification of new treatments and sources of antimicrobial agents is of utmost urgency. An important strategy for tackling bacterial resistance involves the utilization of drug combinations, and natural products derived from plants hold significant potential as a rich source of bioactive compounds that can act as effective adjuvants. This study, therefore, aimed to assess the antibacterial potential and the chemical composition of *Miconia albicans,* a Brazilian medicinal plant used to treat various diseases.

**Methods:**

Ethanolic extracts from leaves and stems of *M. albicans* were obtained and subsequently partitioned to give the corresponding hexane, chloroform, ethyl acetate, and hydromethanolic phases. All extracts and phases had their chemical constitution investigated by HPLC–DAD-MS/MS and GC–MS and were assessed for their antibiofilm and antimicrobial efficacy against *Staphylococcus aureus.* Furthermore, their individual effects and synergistic potential in combination with antibiotics were examined against clinical strains of both *S. aureus* and *Acinetobacter baumannii*. In addition, 10 isolated compounds were obtained from the leaves phases and used for confirmation of the chemical profiles and for antibacterial assays.

**Results:**

Based on the chemical profile analysis, 32 compounds were successfully or tentatively identified, including gallic and ellagic acid derivatives, flavonol glycosides, triterpenes and pheophorbides. Extracts and phases obtained from the medicinal plant *M. albicans* demonstrated synergistic effects when combined with the commercial antibiotics ampicillin and ciprofloxacin, against multi-drug resistant bacteria *S. aureus* and* A. baumannii*, restoring their antibacterial efficacy. Extracts and phases also exhibited antibiofilm property against *S. aureus*. Three key compounds commonly found in the samples, namely gallic acid, quercitrin, and corosolic acid, did not exhibit significant antibacterial activity when assessed individually or in combination with antibiotics against clinical bacterial strains.

**Conclusions:**

Our findings reveal that *M. albicans* exhibits remarkable adjuvant potential for enhancing the effectiveness of antimicrobial drugs against resistant bacteria.

**Supplementary Information:**

The online version contains supplementary material available at 10.1186/s12906-023-04147-w.

## Background

Antimicrobial resistance represents a major global public health concern, characterized by the emergence of new traits that lead to the loss of effectiveness of a drug, whether they arise naturally or are acquired [1]. Pathogens previously susceptible to several drugs have increasingly shown resistance profiles, due to the extensive use of antibiotics. As a result, these microorganisms have developed several defense mechanisms against the effectiveness of drugs [2], causing *c.* 5 million deaths annually, which are directly associated to untreatable resistant infections [1, 3]. Therefore, new treatments are urgently needed to deal with multi-drug resistant bacteria (MDR), and, in this scenario, plants can provide a valuable source of bioactive molecules.

Phytochemicals, or plant natural products, are compounds produced by plants that perform several functions, including protection and adaptation of the species to the environment. These molecules have a wide structural diversity and are produced aiming at biological targets, being, therefore, excellent candidates in the search for bioactive compounds, including new antimicrobial agents [4]. Natural products have demonstrated different mechanisms of antimicrobial action, such as promoting cell wall rupture and lysis, inhibiting biofilm formation, preventing cell wall construction, interrupting microbial DNA replication, and inhibiting the synthesis of bacterial toxins to the host, among others [5, 6]. Furthermore, the remarkable activity of phytochemicals against bacterial virulence factors reinforces the potential of plant natural products in the development of complementary treatments for infectious diseases [7]. The combination of natural products with antibiotics has been recognized as an important strategy to enhance the therapeutic effects of drugs and to limit microbial resistance, mainly by re-sensitizing MDR bacteria to antibiotics, besides preventing the spread of antibiotic resistance [8, 9].

Due to the coexistence of plants and microorganisms, most of these phytochemicals exhibits weak antibiotic activity when evaluated individually, and their potency is several orders of magnitude lower than that of common antibiotics produced by bacteria and fungi. However, plants produce a wide variety of compounds and show successful defense mechanisms, generally employing synergism between two or more molecules as a mechanism to combat pathogens and infections [10].

A synergistic effect occurs when two or more chemical compounds are combined to treat a pathology and, as a result, the combined biological activity of these compounds is greater than the sum of their individual biological activities [11, 12]. The use of natural products in synergistic combinations usually occurs through multi-target actions, mostly by inhibiting or suppressing antibiotic resistance, which frequently leads to the utilization of concentrations below the minimum inhibitory concentration (MIC) [13, 14].

*Miconia albicans* (Sw.) Steud. (Melastomataceae), a shrub distributed in the Brazilian Cerrado and popularly known as 'canela-de-velho', is widely used in folk medicine for treatment of intestinal diseases, infections, arthritis, arthrosis and various inflammations [15]. Antioxidant [16, 17], anxiolytic- and anticonvulsant-like effects [18], antidiabetic [19], antimutagenic [20], anti-hyperalgesic and anti-inflammatory [15] activities have also been reported for this species [21]. Despite possessing several notable biological properties, which justifies its popular use, *M. albicans* has been poorly investigated for its antimicrobial potential.

In this work, the extracts and phases obtained from the leaves and stems of the Brazilian medicinal plant *M. albicans* were evaluated for their antimicrobial potential targeting two strains of the most significant and currently encountered clinical MDR bacteria, namely, *Acinetobacter baumannii* and MRSA, including their individual effects as well as their combined efficacy with antibiotics. Additionally, they were assessed for their ability to inhibit biofilm formation by *S. aureus*. Chemical profiles of the bioactive extracts and phases, analyzed by GC-MS and LC–MS, were also investigated.

## Materials and methods

### General experimental procedures

^1^H and ^13^C NMR spectra were obtained in CDCl_3_ or CD_3_OD (Cambridge Isotope Laboratories) on a Bruker DPX-300 spectrometer (Bruker) operating at 300.13 MHz (^1^H)/75.47 MHz (^13^C). Column chromatography procedures were performed on silica gel 60 (70 − 230 mesh, Merck, Germany) and Sephadex LH-20 (Sigma-Aldrich, USA). Gallic acid was purchased from Sigma Aldrich (Sigma-Aldrich, USA). HPLC with diode-array detection and tandem mass spectrometry (HPLC–DAD-MS/MS) was performed using an LC-DAD-HRESIMS system equipped with a SIL-20A autosampler, a DGU-20A3r vacuum degasser, a thermostated CTO-20A column compartment, and an LC-20AD pump, coupled to an SPD-M20A DAD (all Shimadzu, Japan) and a micrOTOF Q-III high-resolution time-of-flight mass spectrometer (Bruker Daltonics, USA) with an electrospray ionization (ESI) ion source, operating in positive and negative ion modes (120 − 1200 Da and collision energy 45 − 65 V). GC–MS analysis was performed using a Shimadzu GC–MS QP-2010 PLUS Gas Chromatograph (Shimadzu Corporation, Japan) coupled to a mass spectrometer operating at 70 eV, equipped with an autosampler AOC-20i (Shimadzu).

### Plant material

Leaves and stems of *Miconia albicans* (Sw.) Steud were collected from Cerrado region, (Município de Rochedo, MS, Brasil; coordinates: 19°52′32.9" S e 54°48′31.7"W), in February 2019. License for research on Brazil’s biodiversity #A5B7329, issued by National System for the Management of Genetic Heritage and Associated Traditional Knowledge (SISGEN/Brazil). The plant was identified by Professor Dr. Geraldo Alves Damasceno Junior (Institute of Biosciences, Universidade Federal de Mato Grosso do Sul), and a voucher specimen (n^o^ CGMS 74186) was deposited at the CGMS Herbarium of the Universidade Federal de Mato Grosso do Sul.

### Extraction and isolation

Air-dried and powdered leaves (1.6 kg) and stems (1.1 kg) of *M. albicans* were extracted at room temperature with EtOH, yielding 82 g of leaves ethanol extract (LEE) and 19 g of stems ethanol extract (SEE). After concentration *in vacuo*, the residues obtained from the EtOH extracts were partitioned between MeOH–H_2_O 9:1 and hexane, and between MeOH–H_2_O 1:1 and CHCl_3_, followed by ethyl acetate, to give the corresponding leaves (L) and stem (S) phases: hexane (LHxP 8.3 g and SHxP 3.1 g), chloroform (LCP 4.9 g and SCP 0.7 g), ethyl acetate (LEP 12.9 g and SEP 9.1 g), and hydromethanolic (LHP 20.3 g and SHP 1.8 g) phases.

Additional chromatographic separations were performed on LCP and LEP to provide reference compounds for the chemical profile analysis. An aliquot of LCP phase (2.6 g) was chromatographed on a silica gel 70–230 mesh column, using step gradient elution with hexane, hexane–EtOAc (1:1), and EtOAc to give 5 fractions (A → E). Fraction C (hexane–EtOAc 1:1, 16.0 mg) yielded compounds **29** and **31** as a mixture. The LEP phase was chromatographed on a silica gel 70–230 mesh column, using hexane, hexane–EtOAc (3:1, 1:1, 1:3), EtOAc, and EtOAc–MeOH (9:1, 3:1, 1:1) as eluents, to furnish 13 fractions (A → M). Fraction A and B (hexane 100%) yield a mixture of **23** and **25**, and compound **28**, respectively, the latter was also obtained as a white solid precipitate from SHxP. Fraction E (hexane–EtOAc 8:2) gave **18** (63.0 mg), fraction G (hexane–EtOAc 1:3) yielded **19** (3.0 mg), while fractions I and J afforded **20** (30.0 mg) and **21** (40.0 mg), respectively. Fraction H was chromatographed on a Sephadex LH-20 column in MeOH to furnish 9 subfractions (I-IX). Subfraction V gave **10** (10.0 mg).

### Chemical profiles of extracts and phases of *Miconia albicans*

#### HPLC–MS/MS analysis 

Aliquots of the extracts and phases of *M. albicans* (10 mg each) were separately dissolved in 10 mL of MeOH − H_2_O (1:1) and filtered through 0.22 μm PVDF membranes (Allcrom, Brazil). Subsequently, 10 μL aliquots of each solution were separately injected into a Luna RP-18 column [5.0 µm; 150 × 2.0 mm; Phenomenex™ Luna PFP (2), USA] coupled to a sub-2 Security Guard Ultra Cartridge for C18 HPLC and a core − shell column (2.1 mm, Phenomenex, USA). Column temperature was maintained at 50 °C, and the mobile phase, at a flow of 0.2 mL/min, consisted of a linear gradient of water (solvent A) and methanol (solvent B), both containing 0.1% (v/v) of formic acid, as follows: 3% B (0 − 2 min), 3 − 25% B (2 − 25 min), 25 − 80% B (25 − 40 min), 80% B (40 − 43 min), followed by washing and reconditioning of the column (80% B → 3% B, 8 min). The DAD acquisitions were performed in the range of 240 − 800 nm. The data obtained were subsequently processed and analyzed using the DataAnalysis® software version 4.2 (Bruker).

#### *GC–MS analysis*

Hexane phases (leaves and stems) and the isolated compounds (**23, 25, 27–29** and **31**) were dissolved in dichloromethane (1 mg/mL), and injected in a Rtx™-5MS Restek fused silica capillary column (5%-diphenyl–95%- dimethylpolysiloxane, Restek, USA) of 30 m × 0.25 mm i.d., 0.25 µm film thickness. An injection volume of 1 μL was employed, with a split ratio of 1:50. Injector temperature was 250 °C, with the carrier gas (Helium 99.999% purity) at a flow rate of 1 mL/min, and pressure of 87.1 kPa. The oven temperature was programmed from 50 °C (isothermal for 1.5 min), with an increase of 3 °C/min, to 260 °C, ending with a 5 min isothermal at 260 °C. The data were processed in a GCMS Postrun Analysis Software (Shimadzu Corporation, Kyoto, Japan). Triterpenes and sterols were identified by comparing the relative retention (RR) of the samples with the RR of the standard compounds.

### Antimicrobial susceptibility assays

All reagents and media for the antibacterial assays were purchased from Sigma Aldrich™. The reference bacterial strains *Staphylococcus aureus* (ATCC 25904) and *Escherichia coli* (NEWP0022) were purchased from Newman™ and NEWPROV™ Companies, respectively. Clinical *S. aureus* (from human intra-abdominal fluid, β-lactamase producer, *mecA* mediated methicillin resistance), and clinical *Acinetobacter baumannii* (from blood culture, resistant to amikacin, cefepime, ceftazidime, ceftriaxone, ciprofloxacin, imipenem, levofloxacin, meropenem, piperacillin-tazobactam and trimethoprim-sulfamethoxazole) were provided by the Center of Clinical Analysis of the University Hospital, Universidade Federal de Mato Grosso do Sul (Campo Grande-MS, Brazil). The antimicrobial activity was determined by broth microdilution method, as described by Manda et al. (2018) [22]. Initially, samples were solubilized in dimethyl sulfoxide (DMSO). Two-fold dilutions were performed in 96-well plates prepared with Mueller–Hinton broth to reach a final concentration of 4000 μg/mL to 31.3 μg/mL, with a 100 μL final volume in each well for plant extracts and phases. The isolated compounds (**3, 10** and **18**) were evaluated at concentrations ranging from 2000 to 15.6 μg/mL. For ampicillin the concentration ranged from 2000 to 15.6 μg/mL, and for ciprofloxacin from 100 to 0.78 μg/mL. Gentamicin was used as a positive control (60.0 to 0.5 μg/mL). The inoculums were overnight cultures of each bacterial species in Mueller–Hinton agar diluted in sterile saline solution (0.9%) to a cell density of approximately 10^8^ CFU/mL (0.5 in McFarland scale), measured in a MS Tecnopon MCF-500 McFarland turbidimeter. This solution was diluted 1/10 in saline solution (0.9%) and 5 μL were added to each well containing the test samples. All experiments were performed in triplicate and the microdilution trays were incubated at 36ºC for 18 h. Then, 20 μL of an aqueous solution (0.5%) of triphenyltetrazolium chloride (TTC) were added to each well and the trays were again incubated at 36ºC for 2 h. In those wells where bacterial growth did occur, TTC changed from colorless to red. MIC was defined as the lowest concentration of each substance at which no color change occurred and was expressed in μg/mL. The culture medium was used as a negative control, and DMSO was used as a blank.

Synergistic interactions were evaluated using the checkerboard microtiter test, following the method described by Solarte et al. (2017) [23]. Serial two-fold dilutions of the extracts and phases were made vertically in 96-well plates prepared with Mueller–Hinton broth, to reach a concentration of 4000 μg/mL to 31.3 μg/mL, with a 50 μL final volume in each well. For the isolated compounds, the concentrations ranged from 2000 to 15.6 μg/mL. Aliquots (50 μL) of antibiotics solutions in Mueller–Hinton broth were added in each well, so the final concentrations varied horizontally from 100 to 0.05 μg/mL. For the assays with isolated compounds, the concentration of ampicillin ranged from 2000 to 0.98 μg/mL. Bacterial inoculums were prepared as mentioned above, and 5 μL were added to each well containing the test samples, then the plates were incubated at 36ºC for 18 h. After addition of TTC, MICs of the combinations were accessed, and fractional inhibitory concentration (FIC) and fractional inhibitory concentration index (FICI) were calculated by the formulae:

$$\mathrm{FIC}=\mathrm{Combined\;MIC\;of\;the\;sample\;or\;antibiotic}/\mathrm{Individual\;MIC\;of\;the\;sample\;or\;antibiotic}$$

$$\mathrm{FICI}=\mathrm{FIC\;of\;the\;sample}+\mathrm{FIC\;of\;antibiotic}$$

The FICI value was interpreted as: synergism (FICI ≤ 0.5), additive (0.5 < FICI ≤ 1), indifferent (1 < FICI ≤ 4), and antagonist (FICI > 4) [24–26].

### Antibiofilm assay

*Staphylococcus aureus* ATCC 25904 (Newman) was grown on Muller-Hinton agar at 37ºC overnight. Bacterial suspensions with an optical density corresponding to 0.150 (approximately 3 × 10^8^ CFU/mL) at 600 nm (OD 600) were prepared in sterile 0.9% NaCl solution and used in the assays.

Extracts and fractions of *M. albicans* were solubilized in DMSO at 6.25 and 25 mg/mL, providing a final tested concentration of 125 and 500 μg/mL, respectively. *S. aureus* biofilm formation was evaluated using crystal violet technique and planktonic bacterial growth was assessed by the difference between the OD 600 absorbance measured at the end and the beginning of incubation time. All assays were developed in 96-well microtiter plates of polystyrene, as described by Trentin et al. (2011) [27]. As negative control, the samples were replaced by 2% DMSO, and this condition was considered as 100% biofilm formation and bacterial growth, being used to compare the activity of samples. As positive control, samples were replaced by vancomycin (8 μg/mL).

All antibiofilm assays were performed at least in triplicate. The data were analyzed by Student's t-test in relation to the negative control (untreated samples) and a *p*-value ≤ 0.05 was considered to be significant.

## Results

Leaves (L) and stems (S) of *M. albicans* were macerated with ethanol to obtain the respective ethanol extracts of leaves (LEE) and stems (SEE). The EtOH extracts were subsequently partitioned between different solvents to give the corresponding hexane (LHxP and SHxP), CHCl_3_ (LCP and SCP), ethyl acetate (LEP and SEP) and hydromethanol (LHP and SHP) phases.

To investigate the antibacterial properties of *M. albicans*, the foregoing extracts, phases, and three isolated compounds present in most of these samples [gallic acid (**3**), quercitrin (**10**) and corosolic acid (**18**)] were assessed against reference strains of *Staphylococcus aureus* and *Escherichia coli*, and against clinical isolates of methicillin-resistant *S. aureus* (MRSA) and MDR *Acinetobacter baumannii*, both individually and in combination with antibiotics. Furthermore, the antibiofilm potential of extracts and their respective phases were evaluated against *S. aureus*. Antibiofilm properties of **3**, **10** and **18** were not evaluated due to their limited amount.

### Antimicrobial evaluations

As depicted in Table 1, SEE extract demonstrated moderate activity against the reference strain of *S. aureus* and MDR *A. baumannii*, with MIC values of 500 μg/mL, while LEE proved inactive against both strains (MIC = 1000 μg/mL). Both extracts were also inactive against *E. coli* and MRSA. Most of the phases resulting from the liquid–liquid partition of the bioactive extracts proved ineffective against *E. coli* and *S. aureus*, except for LEP, LHP, and SHP, which were moderately active against the Gram-positive reference bacterium. Regarding MRSA and MDR *A. baumannii*, the best results were observed for the SEP phase against both strains and LEP phase against *A. baumannii* strain (MIC values of 500 μg/mL).

**Table 1:** MIC values of *M. albicans* extracts, phases, and isolated compounds against bacterial strains

Sample	MIC (µg/mL)			
	***S. aureus***	***E. coli***	**MRSA**^**a**^	***A. baumannii***^**b**^
LEE	1000	≥ 1000	1000	1000
LHxP	≥ 2000	≥ 1000	≥ 2000	≥ 2000
LEP	500	≥ 1000	1000	500
LCP	1000	≥ 1000	1000	1000
LHP	500	≥ 1000	1000	1000
SEE	500	≥ 1000	1000	500
SHxP	1000	≥ 1000	≥ 2000	1000
SEP	1000	≥ 1000	500	500
SCP	1000	≥ 1000	1000	1000
SHP	500	≥ 1000	1000	1000
Gallic acid (**3**)	31.3	250	250	62.5
Quercitrin (**10**)	≥ 1000	≥ 500	≥ 500	≥ 250
Corosolic acid (**18**)	62.5	≥ 500	≥ 500	≥ 250
Ampicillin	15	≤ 0.5	> 2000	> 2000
Ciprofloxacin	≤ 0.5	≤ 0.5	NT	100
Gentamicin	≤ 0.5	≤ 0.5	15	0.98

*LEE* leaves-ethanol extract, *LHxP* leaves-hexane phase, *LEP* leaves-ethyl acetate-phase, *LCP* leaves-chloroform phase, *LHP* leaves-hydromethanolic phase, *SEE* stems-ethanol extract, *SHxP* stems-hexane phase, *SEP* stems-ethyl acetate phase, *SCP* stems-chloroform phase, *SHP* stems-hydromethanolic phase.^a^Clinical strain, resistant to penicillin G and oxacillin. ^b^Clinical strain, resistant to aminoglycosides, quinolones, 3^rd^ and 4^th^ generation cephalosporins, penicillins, carbapenems, piperacillin-tazobactam and trimethoprim – sulfamethoxazole. *NT* not tested

For reference strain *E. coli*, the isolated compounds **3**, **10** and **18** were ineffective (MIC values ≥ 250) [6], while for *S. aureus*, only compounds **3** and **18** showed moderate activity (MIC values of 31.3 and 62.5 µg/ml, respectively). When evaluated against clinical bacteria, isolated compounds showed moderate to weak activity profiles, with MIC values ranging from ≥ 250 to 62.5 µg/ml against *A. baumannii* and from ≥ 500 to 250 µg/ml against MRSA.

### Antibiofilm activity

**Fig. 1:**
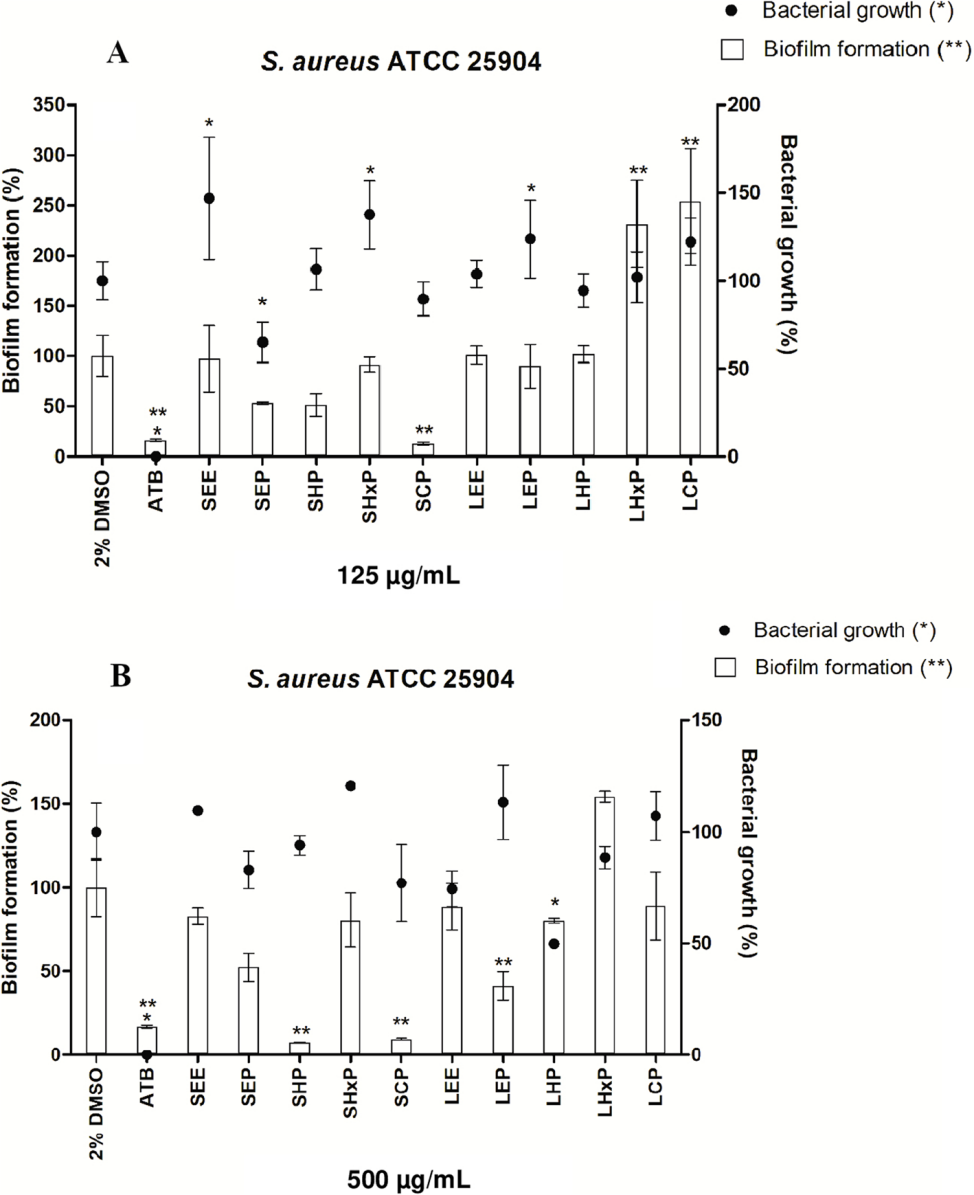
Biofilm formation by *S. aureus* ATCC 25904 treated with extracts and phases of *Miconia albicans*. **A** 125 µg/mL and **B** 500 µg/mL. Significant differences in comparison to negative control (2% DMSO) with p value ≤ 0.05 are represented by ** for biofilm formation and * for bacterial growth

Both Gram-positive and Gram-negative bacteria have the ability to adhere and develop biofilms, but staphylococcal species are most commonly associated with biofilm-related infections, accounting for approximately two-thirds of cases involving indwelling medical devices [28]. Taking this under consideration, and based on the previously observed antimicrobial activity (Table 1), we selected *S. aureus*, a well-known biofilm-forming strain [7, 29], to assess the potential of extracts and phases obtained from *M. albicans* in preventing biofilm formation (Fig. 1). The evaluated extracts LEE and SEE did not exhibit any significant activity against biofilm formation. However, upon fractionation, the stems fractions revealed the highest antibiofilm activity against *S. aureus.* Among these fractions, SCP, SHP and SEP particularly stood out for their effectiveness.

At the highest concentration evaluated (500 μg/mL), except for LHxP, all samples inhibited biofilm formation. In this condition, the most active phases, for which *S. aureus* biofilm formation was suppressed, were SHP, SCP and LEP (allowing only 7.6 ± 1.4%, 9 ± 5.3%, and 41 ± 20% of biofilm formation, respectively) (Fig. 1).

At a concentration of 125 µg/mL, both SHP and SCP exhibited sustained activity, with SCP showing a particularly high rate of biofilm inhibition, by allowing only 13 ± 10% of biofilm formation to occur. It is worth noting that SEP maintained the inhibition rate of *c.* 50% at both tested concentrations. It is important to highlight that the most active phases, at the tested concentrations, did not exhibit any interference with bacterial growth (Fig. 1).

### Synergistic effect

Extracts and phases were further assessed against clinical MRSA and MDR *A. baumannii* strains in combination with antibiotics currently used in therapy, ampicillin and ciprofloxacin, to which these bacteria have already shown to be resistant.

As depicted in Table 2, combinations of *M. albicans* extracts and phases with ampicillin revealed a synergistic effect against MRSA, except for LCP, LHxP and LEP, which showed an additive effect. Promising results were observed for the LEE extract, with a Fractional Inhibitory Concentration Index (FICI) value of 0.04, and a reduction of 32-fold in the LEE MIC value. Significant results were also observed for all extracts and phases from stems, particularly emphasizing the effectiveness of the SCP and SHP phases, both with FICI of 0.08, followed by the SEP phase and the SEE extract, with FICIs values of 0.13 and 0.14, respectively.

**Table 2:** Fractional inhibitory concentration (FIC) and FIC indices (FICI) of antibiotic-extracts/ phases pairs, against MRSA

MRSA *Staphylococcus aureus*^a^				
**Sample/Combination**	**Combined MIC** **(µg/mL)**	**FIC**	**FICI**	**Result**
SEE + AMP			0.14	Synergism
SEE	125	0.125		
AMP	25	0.0125		
SHxP + AMP			0.26	Synergism
SHxP	500	0.25		
AMP	25	0.0125		
SEP + AMP			0.13	Synergism
SEP	62.5	0.125		
AMP	12.5	0.0063		
SCP + AMP			0.08	Synergism
SCP	62.5	0.0625		
AMP	25	0.0125		
SHP + AMP			0.08	Synergism
SHP	62.5	0.0625		
AMP	25	0.0125		
LEE + AMP			0.04	Synergism
LEE	31.3	0.031		
AMP	25	0.0125		
LHxP + AMP			0.51	Additivity
LHxP	1000	0.5		
AMP	12.5	0.0063		
LEP + AMP			0.51	Additivity
LEP	1000	0.5		
AMP	12.5	0.0063		
LCP + AMP			1	Additivity
LCP	1000	1		
AMP	12.5	0.0063		
LHP + AMP			0.26	Synergism
LHP	250	0.25		
AMP	25	0.0125		
**3** + AMP			2	Indifference
**3**	250	1		
AMP	> 2000	1		
**10** + AMP			2	Indifference
**10**	≥ 500	1		
AMP	> 2000	1		
**18** + AMP			2	Indifference
**18**	≥ 500	1		
AMP	> 2000	1		

^a^Clinical strain, resistant to penicillin G and oxacillin. *LEE* leaves-ethanol extract, *LHxP* leaves-hexane phase, *LEP* leaves-ethyl acetate phase, *LCP* leaves-chloroform phase, *LHP* leaves-hydromethanolic phase, *SEE* stems-ethanol extract, *SHxP* stems-hexane phase, *SEP* stems-ethyl acetate phase, *SCP* stems-chloroform phase, *SHP* stems-hydromethanolic phase, *AMP* Ampicillin. For FIC calculation, MIC values of LHxP and SHxP were considered = 2000 µg/mL

When evaluated in combination with antibiotics against MDR *A. baumannii*, the extracts and phases demonstrated a notable enhancement in the activity of both ampicillin and ciprofloxacin (Table 3). All tested combinations showed synergism or additive effects, except for the combination of SEE and ampicillin, resulting in FICI = 2, indicating indifference.

**Table 3:** Fractional inhibitory concentration (FIC) and FIC indices (FICI) of antibiotic-extracts/ phases pairs, against *A. baumannii*

*Acinetobacter baumannii*^a^									
**Ampicillin**					**Ciprofloxacin**				
**Sample/Combination**	**Combined MIC** **(µg/mL)**	**FIC**	**FICI**	**Result**	**Sample/Combination**	**Combined MIC** **(µg/mL)**	**FIC**	**FICI**	**Result**
SEE + AMP			2	Indifference	SEE + CIP			0.625	Additivity
SEE	1000	2			SEE	250	0.5		
AMP	3.125	0.0016			CIP	12.5	0.125		
SHxP + AMP			0.51	Additivity	SHxP + CIP			0.27	Synergism
SHxP	500	0.5			SHxP	250	0.25		
AMP	12.5	0.0063			CIP	1.56	0.016		
SEP + AMP			0.51	Additivity	SEP + CIP			0.25	Synergism
SEP	250	0.5			SEP	125	0.25		
AMP	12.5	0.0063			CIP	0.195	0.002		
SCP + AMP			0.5	Synergism	SCP + CIP			0.25	Synergism
SCP	500	0.5			SCP	125	0.125		
AMP	1.56	7.8 × 10^–4^			CIP	12.5	0.125		
SHP + AMP			0.5	Synergism	SHP + CIP			0.5	Synergism
SHP	500	0.5			SHP	500	0.5		
AMP	0.049	2.5 × 10^–5^			CIP	0.05	5 × 10^–4^		
LEE + AMP			0.51	Additivity	LEE + CIP			0.25	Synergism
LEE	500	0.5			LEE	125	0.125		
AMP	25	0.0125			CIP	12.5	0.125		
LHxP + AMP			0.28	Synergism	LHxP + CIP			0.13	Synergism
LHxP	500	0.25			LHxP	250	0.125		
AMP	50	0.025			CIP	0.39	0.004		
LEP + AMP			0.55	Additivity	LEP + CIP			0.31	Synergism
LEP	250	0.5			LEP	31.3	0.06		
AMP	100	0.05			CIP	25	0.25		
LCP + AMP			0.28	Synergism	LCP + CIP			0.25	Synergism
LCP	250	0.25			LCP	250	0.25		
AMP	50	0.025			CIP	0.39	0.004		
LHP + AMP			0.503	Additivity	LHP + CIP			0.38	Synergism
LHP	500	0.5			LHP	250	0.25		
AMP	6.25	0.003			CIP	12.5	0.125		
**3** + AMP			2	Indifference	**3** + CIP			2	Indifference
**3**	62.5	1			**3**	62.5	1		
AMP	> 2000	1			CIP	100	1		
**10** + AMP			2	Indifference	**10** + CIP			2	Indifference
**10**	≥ 250	1			**10**	≥ 250	1		
AMP	> 2000	1			CIP	100	1		
**18** + AMP			2	Indifference	**18** + CIP			2	Indifference
**18**	≥ 250	1			**18**	≥ 250	1		
AMP	> 2000	1			CIP	100	1		

^a^Clinical strain, resistant to aminoglycosides, quinolones, 3^rd^ and 4^th^ generation cephalosporins, penicillins, carbapenems, piperacillin-tazobactam and trimethoprim – sulfamethoxazole. *LEP* leaves-ethyl acetate phase, *LCP* leaves-chloroform phase, *LHP* leaves-hydromethanolic phase, *SEE* stems-ethanol extract, *SHxP* stems-hexane phase, *SEP* stems-ethyl acetate phase, *SCP* stems-chloroform phase, *SHP* stems-hydromethanolic phase, *AMP* Ampicillin, *CIP* Ciprofloxacin. For FIC calculation, MIC values of LHxP and SHxP were considered = 2000 µg/mL

For the combination with ampicillin, the best activity profiles were observed for extracts and phases from leaves, especially for the non-polar phases LCP and LHxP, with FICI 0.28.

The most promising results against *A. baumannii* were observed for the combination of extracts and phases with the antibiotic ciprofloxacin. Except for the additive effect observed for the SEE extract, all other combinations showed a synergistic effect, with FICI values ranging from 0.13 to 0.5 (Table 3).

The isolated compounds **3**, **10** and **18** did not show additive or synergistic effects (Tables 2 and 3).

### Chemical profile

**Fig. 2:**
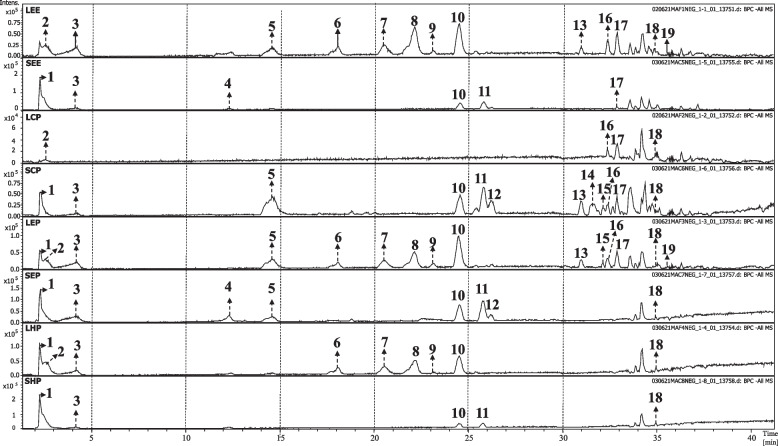
HPLC–DAD-MS/MS (negative mode) profiles of ethanol extracts and phases of *M. albicans*. Peak numbers refer to compounds listed in Table 4. LEE = Leaves-ethanol extract. LCP = Leaves-chloroform phase. LEP = Leaves-ethyl acetate phase. LHP = Leaves-hydromethanolic phase. SEE = Stems-ethanol extract. SCP = Stems-chloroform phase. SEP = Stems-ethyl acetate phase. SHP = Stems-hydromethanolic phase

The chemical profiles of *M. albicans* extracts (LEE and SEE) and their respective phases were determined by HPLC–UV-MS (Fig. 2, Table 4), in which 21 metabolites were tentatively identified, based on retention time, online UV, and HRESIMS data, including mass spectrometric fragmentation patterns (MS and MS2). These data were compared with those of authentic standards, literature data, or both (Table 4).

**Table 4:** HPLC–UV-MS data of annotated compounds from extracts and phases of *M. albicans* leaves and stems. + : presence; -: absence

N^o^	R_t_ (min)	Molecular formula	Parent ion (*m/z*)	Product ion (*m/z*) (Relative intensity %)	Compound	Distribution among sample							
						**LEE**	**LCP**	**LEP**	**LHP**	**SEE**	**SCP**	**SEP**	**SHP**
**1**	2.3	C_24_H_44_O_22_	683.2281 [M–H]^–^	341 (100) [M-C_12_H_22_O_11_]^−^; 179 (10) [M-H-C_6_H_10_O_5_]^−^	Tetramer of hexose	–	–	+	+	+	+	+	+
**2**	2.6	C_7_H_12_O_6_	191.0565 [M–H]^–^	173 (42) [M–H^–^H_2_O]^–^	Quinic acid	+	+	+	+	–	–	–	–
**3**	4.2	C_7_H_6_O_5_	169.0133 [M–H]^–^	125 (10) [M–COOH]^–^	Gallic acid^a^	+	–	+	+	+	+	+	+
**4**	12.3	C_20_H_22_O_12_	453.1051 [M-H]^−^	169 (100) [gallate moiety]; 124 (85)	Gallic acid derivative	–	–	–	–	+	–	+	–
**5**	14.7	C_9_H_10_O_5_	197.0453 [M–H]^–^	169 (34) [M–CH_2_CH_3_]; 124 (8) [M–COOCH_2_CH_3_]^–^	Ethyl gallate	+	–	+	+	–	+	+	–
**6**	18.1	C_26_H_34_O_12_	537.1985 [M–H]^–^	271 (14); 211 (29); 169 (83) [gallate moiety]; 151 (14) [galloyl moiety]	Gallic acid derivative	+	–	+	+	–	–	–	–
**7**	20.5	C_28_H_24_O_16_	615.1001 [M–H]^–^	301 (32) [Y_0_]^–^; 300 (100) [Y_0_–H]^–^; 271 (15) [Y_0_–CH_2_O]^–^; 255 [Y_0_–CO-H_2_O]^–^ (9); 169 (8) [gallate moiety]	Quercetin-*O*-galloyl-hexoside	+	–	+	+	–	–	–	–
**8**	22.1	C_21_H_20_O_12_	463.0908 [M–H]^–^	301 (11) [Y_0_]^–^; 300 (37) [Y_0_–H]^–^; 271 (100) [Y_0_–CH_2_O]^–^; 255 [Y_0_–CO-H_2_O]^–^; 151 (7) [^1,3^A_0_–H]^–^	Quercetin-*O-*hexoside (Isoquercitrin)	+	–	+	+	–	–	–	–
**9**	23.1	C_20_H_18_O_11_	433.0799 [M–H]^–^	300 (10) [Y_0_–H]^–^; 271 (100) [Y_0_–CH_2_O]^–^; 255 (28) [Y_0_–CO-H_2_O]^–^;	Quercetin-*O-*pentoside	+	–	+	+	–	–	–	–
**10**	24.6	C_21_H_20_O_11_	447.0929 [M–H]^–^	301 (4) [Y_0_]^–^; 300 (18) [Y_0_–H]^–^; 271 (100) [Y_0_–CH_2_O]^–^; 255 (54) [Y_0_–CO-H_2_O]^–^; 151 (8) [^1,3^A_0_–H]^–^	Quercetin-*O-*deoxyhexoside (quercitrin)^a^	+	–	+	+	+	+	+	+
**11**	25.8	C_21_H_18_O_12_	461.0731 [M–H]^–^	315 (37) [Y_0_–CH_2_O]^–^; 299 (100) [Y_0_–CO-H_2_O]^–^;	Isorhamnetin-*O*-deoxyhexoside	–	–	–	–	+	+	+	+
**12**	26.2	C_21_H_18_O_12_	461.0741 [M–H]^–^	313 (66) [M-H-CH_3_-pentosyl]; 298 (100) [M-H-2CH_3_-pentosyl]^–^; 285 (20); 269 (68)	di-*O*-methyl-*O*-pentosyl ellagic acid	–	–	–	–	–	+	+	–
**13**	31.0	C_16_H_10_O_8_	329.0316 [M–H]^–^	270 (79) [M − H − 2CH_3_ − CO]^−^, 242 (100); 215 (40); 187 (32); 171 (15); 159 (11); 143 (5)	di-*O*-methyl ellagic acid	–	–	+	–	–	+	–	–
**14**	31.7	C_30_H_48_O_6_	503.3398 [M–H]^–^	485 (90)	Triterpene	–	–	–	–	–	+	–	–
**15**	32.1	C_30_H_48_O_5_	487.3452 [M–H]^–^	–	Triterpene	–	–	+	–	–	+	–	–
**16**	32.4	C_16_H_8_O_8_	327.0155 [M–H]^–^	255 (28); 212 (21); 200 (56); 184 (49); 172 (36); 156 (80); 144 (31)	Ellagic acid derivative (O-methyl-methylenedioxy ellagic acid)	+	+	+	–	–	+	–	–
**17**	32.9	C_17_H_12_O_8_	343.0465 [M–H]^–^	328 (100) [M − H − CH_3_]^−^; 313 (17) [M − H − 2CH_3_]^−^; 298 (15) [M − H − 3CH_3_]^−^; 270 (94) [M − H − CH_3_ − CO]^−^	tri-*O*-methyl ellagic acid	+	+	+	–	+	+	–	–
**18**	35.0	C_30_H_48_O_4_	471.3615 [M–H]^–^	–	Corosolic acid^a^	+	+	+	+	+	+	+	+
**19**	35.6	C_30_H_48_O_3_	455.3656 [M–H]^–^	–	Betulinic acid^a^	+	–	+	–	–	–	–	–
**20**	39.4	C_35_H_36_N_4_O_5_	593.2780 [M + H]^+^	565 (6); 533 (81); 505 (38); 461 (39); 387 (55)	Pheophorbide B^a^	+	+	+	–	–	–	–	–
**21**	40.6	C_37_H_40_N_4_O_5_	621.3160 [M + H]^+^	593 (8); 561 (100); 533 (51); 505 (28)	Pheophorbide A ethyl ester^a^	+	+	+	–	+	+	–	–

^a^ Compounds identified by comparing retention times and MS data with those of reference compounds. *LEE* Leaves-ethanol extract, *LCP* Leaves-chloroform phase, *LEP* Leaves-ethyl acetate phase, *LHP* Leaves-hydromethanolic phase, *SEE* Stems-ethanol extract, *SCP* Stems-chloroform phase, *SEP* Stems-ethyl acetate phase, *SHP* Stems-hydromethanolic phase

The retention time and mass spectrum data, along with the peak assignments for compounds annotated and/or identified (using negative ionization mode for compounds **1**–**19** and positive ionization mode for **20** and **21**, non-ionizable in negative mode) are described in Table 4. The identified metabolites consist of gallic and ellagic acid derivatives, flavonol glycosides, triterpenes and pheophorbides.

Compound **1** was annotated as a tetramer of hexose based on literature data [30], while **2** showed MS/MS compatible to a quinic acid pattern [31]. Compound **3** was assigned to gallic acid, based on its [M–H]^–^ ion at *m/z* 169.0147 and its MS/MS was in accordance with a typical gallic acid pattern [31]. Additionally, results revealed the presence of three gallic acid derivatives, with **5** assigned to ethyl gallate [31], while **4** and **6** were annotated as unknown gallic acid derivatives [32].

Five compounds (**7–11**) were characterized as flavonols, based on the mass spectra of their deprotonated glycosides and the presence of ions corresponding to their deprotonated aglycones at *m/z* 300/301 (for quercetin) and 315 (for isorhamnetin), generated by the loss of the sugar units. Furthermore, fragment ions at *m/z* 271 [Y_0_-H_2_CO]^–^ and 255 [Y_0_–CO-H_2_O^–^]^–^ from fragmentation of quercetin, and *m/z* 315 [Y_0_-CH_2_O]^–^ and 299 [Y_0_–CO-H_2_O]^–^ from fragmentation of isorhamnetin were detected [33–36]. Peak **7** (*m/z* 615.1000, C_28_H_24_O_16_) showed fragment ions at *m/z* 301/300 and 169, compatible with a quercetin hexoside derivative. The fragment ion at 169 Da indicated the presence of a galloyl group, allowing the annotation of **7** as a quercetin-galloyl-hexoside derivative [15].

Compounds **12, 13, 16** and **17** were assigned to ellagic acid derivatives. The [M-H]- ions of **13** and **17** exceeded that of ellagic acid by 28 and 42 Da, corresponding to the extra two and three methyl groups, respectively. Their fragmentation patterns were characterized by a loss of 15 Da [M-CH_3_-H]^–^, attributed to the loss of the methyl radical. These compounds were thus tentatively identified as dimethylellagic and trimethylellagic acids, respectively. Compounds **12** and **16** were annotated as di-*O*-methyl-*O*-pentosyl ellagic acid and methyl ellagic acid bearing a methylenedioxy substituent, respectively [31, 37].

Compounds **18** and **19** showed peaks of deprotonated ions at *m/z* 471.3615 and 455.3656, compatible with molecular formulae C_30_H_48_O_4_ and C_30_H_48_O_3_, respectively. These compounds were identified as the triterpenes corosolic acid (**18**) and betulinic acid (**19**), based on the analysis of their MS data and comparison with reference compounds. Compounds **14** and **15** also presented deprotonated ions at *m/z* 503.3398 and 487.3452, in accordance with molecular formulae of compounds containing 30 carbons, therefore annotated as triterpenes.

Compounds **20** and **21** were observed exclusively in positive ionization mode, with protonated ions at *m/z* 593.2780 and *m/z* 621.3160, compatible with the molecular formulae C_35_H_36_N_4_O_5_ and C_37_H_40_N_4_O_5_, respectively. These compounds were identified as pheophorbide B and pheophorbide A ethyl ester, respectively, based on the analysis of their MS data and retention time, as well as by comparison with data of authentic samples isolated and identified from the same extract, together with literature report [38].

**Fig. 3:**
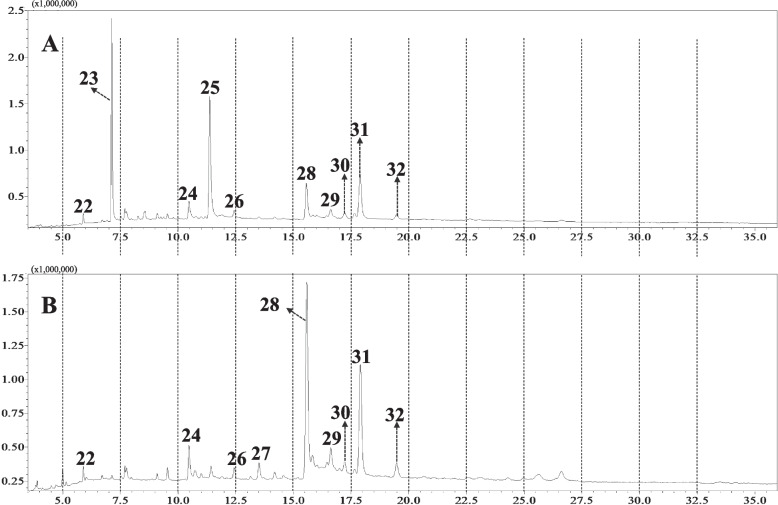
GC–MS profiles of hexane phases of *M. albicans*. Peak numbers refer to compounds listed in Table 5. **A** LHxP: Leaves-hexane phase; **B** SHxP: Stems-hexane phase

The chemical composition of both hexane phases was investigated using gas chromatography coupled to mass spectrometry (GC–MS) **(**Fig. 3**)**. The identification of the constituents was performed by comparing the mass spectra obtained with those of the equipment database (Wiley 7 lib and Nist 08 lib), literature data [39], and by using authentic standards.

Eleven major compounds were identified in the hexane phases (LHxP and SHxP) as belonging to the classes of triterpenes, steroids, fatty alcohol, and tocopherol (Table 5, Fig. 3). The identification of these compounds was carried out by analyzing mass data, retention time, fragmentation pattern, and comparison with data obtained from isolated compounds and published literature [39].

**Table 5:** Main metabolites identified by CG-MS in the hexane phases of *M. albicans.* LHxP: Leaves-hexane phase; SHxP: Stems-hexane phase. + : presence; -: absence

No	R_t_ (min)	Molecular Formula	[M]^+^	Identification	Product ion (*m/z*) (Relative intensity %)	LHxP	SHxP
**22**	5.894	C_21_H_44_	296	Heneicosane	57 (100); 71 (94); 85 (63); 99 (31)	+	+
**23**	7.128	C_30_H_50_	410	Squalene^a^	69 (100); 81 (67); 95 (21)	+	-
**24**	10.475	C_27_H_56_O	396	Heptacosanol	55 (62); 57 (97); 97 (100); 111 (55); 139 (16)	+	+
**25**	11.376	C_29_H_50_O_2_	430	Vitamin E^a^	165 (100); 430 (58)	+	-
**26**	12.436	C_30_H_60_O_2_	452	n.i	97 (100); 111 (57); 71 (55)	+	+
**27**	13.510	C_28_H_48_O	400	Campesterol^a^	55 (61); 81 (83); 95 (88); 107(83); 133 (52); 145 (100); 159 (66); 199 (26); 213 (59); 231 (28); 255 (41); 273 (23); 289 (35); 315 (43); 367 (38); 382 (44)	-	+
**28**	15.566	C_29_H_50_O	414	β-Sitosterol^a^	81 (90); 95 (80); 145 (100); 213 (58); 255 (45); 303 (27); 329 (36); 381 (38)	+	+
**29**	16.624	C_30_H_50_O	426	β-Amyrin^a^	69 (25); 95 (41); 135 (39); 161 (30); 175 (18); 189 (45); 203 (37); 218 (100); 411 (2)	+	+
**30**	17.224	C_30_H_48_O	424	β /α-Amyrone	189 (37); 203 (38); 218 (100)	+	+
**31**	17.893	C_30_H_50_O	426	α-Amyrin^a^	69 (25); 95 (41); 135 (39); 161 (30); 175 (18); 189 (45); 203 (37); 218 (100); 411 (2)	+	+
**32**	19.473	C_29_H_48_O	412	Stigmast-4-en-3-one	81 (20); 95 (24); 107 (20); 124 (100); 147 (25); 149 (22); 229 (38); 289 (17); 370 (6); 398 (3)	+	+

^a^Confirmed with reference compounds. *N.i*. not identified

Compounds **10, 18–21, 23, 25, 28, 29** and **31** were isolated as described on item “Extraction and isolation” (Materials and methods) and identified by analysis of ^1^H and ^13^C NMR data, provided in the Supplementary material.

## Discussion 

The extract and phases of *M. albicans* obtained from stems showed the best activity profile against Gram-positive bacteria, when tested against the reference strain of *S. aureus*, in the inhibition of biofilm formation, and in synergism with antibiotics against MRSA.

In the evaluation of the antimicrobial action, the SEE extract displayed the most potent activity, with a MIC of 500 µg/mL against the reference strain of *S. aureus.* Identical MIC values were also observed for LEP, LHP, and SHP phases. When tested against clinical bacteria, the SEP phase showed the best activity against both resistant clinical strains MRSA and *A. baumannii* (MIC of 500 µg/mL) [6, 40].

The chemical profile of the SEP phase analyzed by HPLC–MS revealed the presence of flavonoids quercitrin (**10**) and isorhamnetin-*O*-deoxyhexoside (**11)**, as well as gallic acid derivatives (**3–5**) as major constituents. In addition to their widely known antioxidant properties, flavonoids and aromatic compounds have shown promising antimicrobial activities [41–43], including their ability to inhibit biofilm formation by *Streptococcus mutans*, such as the antibiofilm activity exerted by quercitrin (**10**) against this microorganism [44]. Antimicrobial activities were previously reported for gallic acid (**3**) against *Escherichia coli* ATCC25922, *Enterococcus faecalis* OS4 and *Salmonella* Typhi MD17 [45], with MIC values of *c.* 200 µg/mL, while ethyl gallate (**5**) inhibited *Shigella dysenteriae* CMCC 51105, *Escherichia coli* ATCC 25922, *Salmonella typhimurium* CMCC 50115, *S. aureus,* and *S. albus* with MIC of 240 µg/mL [46], besides acting in synergy with tetracycline against MRSA [47].

Previous studies on *Miconia* species have reported weak antimicrobial activity of leaf extracts from *M. cabucu, M. stenostachya* e *M. rubuginosa* against *S. aureus*, *S. epidermidis*, *Candida albicans*, *Micrococcus luteus*, *Bacillus subtilis*, and *B. cereus*, with MIC values ranging from 1,500 to 7,500 µg/mL [48]. A similar activity profile was observed for *M. latecrenata*, which inhibited the growth of *S. aureus* and *P. aeruginosa* with MIC values of 300 µg/mL and 2,500 µg/mL, respectively [49].

One of the mechanisms by which commensal bacteria, like *S. aureus*, can establish persistent and challenging infections that are difficult to eradicate is through the formation of bacterial biofilms. The ability of *S. aureus* to form biofilms has led to cases of persistent chronic infections, particularly in host tissues where implanted materials such as valves or catheters are present, resulting in serious conditions like osteomyelitis, endocarditis, and infections in prosthetic joints, pacemakers, and other implanted devices. Bacterial cells enclosed in polymer-based matrix promote an increase in resistance to antibiotics and even immune defense mechanisms, making these infections particularly challenging to treat and eliminate [50].

The antibiofilm potential of *M. albicans* extracts and phases was evaluated against *S. aureus*, in which the stem phases SCP and SHP demonstrated promising antibiofilm inhibitory activity, allowing only 9 ± 5.3% and 7.6 ± 14% of biofilm formation at 500 µg/mL. The SCP phase also stands out for its remarkable antibiofilm activity, particularly for being the only sample to maintain strong efficacy even at the lowest concentration tested (125 µg/mL), allowing the development of only *c.*13% of biofilm formation. Analysis of the chemical composition of SCP revealed a diverse chemical profile, with the presence of ellagic acid (**12**, **13**, **16**, and **17**) and flavonoid (**10** and **11**) derivatives as major constituents, in addition to triterpenes (**14, 15** and **18**). Literature reports antibiofilm properties of some compounds that have been detected in SCP, such as ellagic acid derivatives (glycosides) against *S. aureus* [51]. Previously isolated from *Amphiblemma monticola* (Melastomataceae) and similar to compound **13**, 3,4′-di-*O*-methylellagic acid showed the best anti-staphylococcal activity against MRSA and methicillin-sensitive *S. aureus* (MSSA), with MIC values ranging from 16 to 32 μg/mL [52]. Corosolic acid (**18**) has been reported to exhibit activity against the formation of *P. aeruginosa* biofilm, and showed synergistic effects with ciprofloxacin, enhancing the susceptibility of bacterial biofilm to this antibiotic [53]. In addition, corosolic acid has demonstrated the ability to increase the susceptibility of resistant *Klebsiella pneumoniae* to carbapenem antibiotics [54]. In terms of chemical composition, the SHP phase exhibited the least complex profile among the analyzed samples, and shares compounds **1, 3, 10, 11** and **18** with the SCP phase.

Although several studies have proved the antimicrobial properties of isolated compounds from natural sources, promising results have been obtained for plant extracts in combination to antibiotics as a synergistic approach in combating MDR bacteria [55].

Indeed, antibacterial combination therapies have become an important treatment choice for patients with multidrug resistant bacterial infections in clinical settings. Furthermore, when current antibiotics with a single target are employed, the large dosages needed for effectiveness frequently result in bioavailability issues, undesirable side effects, and the emergence of resistance. High doses of a single product may not be necessary if multiple targets could be achieved using antibacterial adjuvants [6]. Adjuvants provide a complementary and alternative approach to the discovery of novel antibiotics by providing a way to both prevent the development of resistance and restore the effectiveness of already prescribed medications. The criteria for the development of novel antimicrobials are strict and involve the thorough evaluation of efficacy and safety indexes, including the comparison of these indexes with those of the initial successful natural product drugs, primarily derived from microorganisms. In addition, treatment of bacterial infections usually requires doses on the gram scale—much higher than other drugs. Therefore, toxicity and efficacy parameters are tough to match for new molecules, which reinforces the importance of initiatives aiming at preserving the currently existing drugs as much as possible [56].

Biologically active plant-derived products have the ability to combat antibiotic resistance and act in synergism with existing antibiotics. One such example is the essential oil obtained from *Pectis substriata* (Asteraceae), which has demonstrated a synergistic effect when combined with antibiotics against clinical drug-resistant *Staphylococcus warneri*. Additionally, it has shown additive effects against pathogens, such as *S. aureus* and *S. intermedius* [57]. Another example is the antibacterial activity of the dichloromethane extract obtained from the leaves of the shea butter tree (*Vitellaria paradoxa* C.F. Gaertn.—Sapotaceae). This extract combined with ampicillin, oxacillin, and nafcillin exhibited synergism against MRSA, by specifically targeting beta-lactamase enzymes [58]. Ilanko et al. (2019) [59] have studied the antimicrobial properties of *Moringa oleifera* (Moringaceae) extracts (leaf, pulp and seeds extracts) against a panel of bacterial triggers of autoimmune inflammatory diseases, alone and in combination with various antibiotics, achieving good results of synergistic or additive effects. Hence, once natural products with limited antimicrobial activity can act as potential allies in the search for novel antimicrobials through synergistic interactions with commercial antibiotics, the extracts and fractions of *M. albicans* were also assessed in this context.

Methicillin resistant *S. aureus* (MRSA) has become a major nosocomial pathogen [60], ranking among the main etiologic agents of hospital-associated bloodstream infections and nosocomial pneumonia, and representing *c.* 60% of *S. aureus* isolated from hospitalized patients [61]. Clinical cases of persistent infections have increased worldwide, making this microorganism an important target for the search for therapeutic alternatives, particularly crucial for hospitalized patients or those receiving intensive care.

All extracts and phases of *M. albicans* showed synergistic or additive effect with the antibiotic ampicillin against clinical MRSA, with FICI values ranging from 1 to 0.04. In addition to restoring the ampicillin activity, it is noteworthy that the combinations also resulted in a significant reduction in the MIC of this antibiotic, which ranged from approximately 80 to 160-fold. Plants of the same genus also showed synergistic activity against reference strains of *S. aureus* (ATCC29213) by a combination of the organic extract (DCM/MeOH) obtained from the leaves of *M. latecrenata* with ampicillin, with FICI 0.4, and combination of the ethyl acetate phase with tetracycline against *P. aeruginosa* (ATCC 27853), with FICI 0.3 [49]. In general, the combination of SEE and its phases with ampicillin demonstrated greater efficacy against MRSA. However, the best activity was observed for LEE with FICI 0.04, reducing the MIC of this antibiotic by 160-fold, and that of the LEE extract individually by 32-fold. It is noteworthy that LEE presented the most complex chemical profile among the analyzed samples, which revealed the presence of a diverse array of compounds, such as glycosylated flavonoids (**7–10**) as major constituents, in addition to quinic acid (**2),** gallic acid (**3**), ellagic acid derivatives (**13,16- 17**), triterpenes (**18–19**), and pheophorbides (**20–21**).

Antimicrobial activities have already been reported for some of the aforementioned compounds, such as quinic acid (**2**), with a prominent bacteriostatic and bactericidal action against *Escherichia coli* (IFO 3301), with the highest activity against this bacterium achieved by the combination of quinic acid with caffeic acid [62]. Flavonoid glucosides, like compounds **7–11** [63], and their respective aglycones, such as quercetin, are also often reported as membrane-disrupting agents against bacterial cells [42]. A previous review regarding the physicochemical parameters and antibacterial activities of 66 flavonoids against *S. aureus* pointed out that flavonoids primary sites of action on Gram-positive bacteria were in the cell membrane, which probably involved phospholipid bilayer degradation, suppression of the respiratory chain or ATP generation, among other mechanisms [41]. Flavonoids bearing galloyl moieties, such as **7**, were isolated from *Woodfordia uniflora* (Lythraceae) and showed significant antibacterial properties against MRSA, by inhibiting biofilm formation, and also by acting synergistically with methicillin [13]. The antibacterial activity of triterpenes, like **14, 15, 18, 19, 23** and **29–31**, against Gram-positive bacteria has been frequently described. A systematic review on activity of pentacyclic triterpenoids against *S. aureus* reported that their remarkable antistaphylococcal properties are related to modifications on membrane permeability through hydrophobic interaction and accumulation of these compounds in the bacterial membrane [64].

Also deserves attention the adjuvant properties showed by SCP and SHP phases against MRSA, as they present FICI 0.08, indicating a significant reduction of the MIC of ampicillin by 80-fold and of the phases by 16-fold. In addition, these phases showed remarkable antibiofilm activity profiles.

MDR *A. baumannii,* at the top of the WHO list of priorities for the development of novel antimicrobials, is considered a critical microorganism because it is often associated with nosocomial infection outbreaks, with a high incidence in intensive care units [65]. This particular bacterium which has been isolated from hospitals worldwide, including Brazil, is associated with an approximate 30% increase in mortality risk among hospitalized patients infected with *A. baumannii* [66].

In the present study, all combinations of *M. albicans* extracts and phases with ampicillin and ciprofloxacin proved to be active against clinical MDR *A. baumanii*. Contrary to the observations with MRSA, the most promising results in combination with ampicillin were obtained with the phases originating from the leaves of *M. albicans,* namely LHxP and LCP, with FICI values of 0.28, while LEE, LHP and LEP showed additivity in combination with this antibiotic. However, the most effective combination was observed with ciprofloxacin, wherein all samples, except for SEE, acted in synergism, restoring the activity of this drug. Regarding the stem samples in combination with this antibiotic, SEP, SCP and SHxP proved the most active, with FICI values of 0.25, 0.25 and 0.27, respectively. For the combination of leaves samples with ciprofloxacin, FICI values ranged from 0.13 to 0.38, and the best result was achieved for LHxP. It is important to highlight that for carpabenem-resistant *A. baumannii*, such as the clinical strain evaluated in this work, treatment options are limited and also face important pharmacokinetic drawbacks [67].

The chemical profile of LHxP revealed squalene (**23**) as its major constituent (Fig. 3), which was only detected in this phase. This compound, which is regarded as a precursor of triterpenes, has been reported to exhibit antimicrobial activity against *Mycobacterium tuberculosis* H37Ra with MIC of 100 µg/mL [68]. Regarding the triterpene α-Amyrin (**31**), the second most abundant compound in LHxP (Fig. 3), literature describes its antibacterial activity against MSSA and MRSA, with MIC of 64 µg/mL [69].

Analysis of the chemical profile of the LCP phase by HPLC–MS revealed the presence of *O*-methyl-methylenedioxi ellagic acid (**16**) and tri-*O*-methyl ellagic acid (**17**). The di-*O*-methyl-O-pentosyl ellagic acid derivative has shown antibacterial activities against *E. coli* (ATCC25922), *Salmonella typhi* MD17, *Enterococcus faecalis* OS4, *S. aureus,* and *P. mirabilis* (ATCC 7002), with MICs ranging from 19.53 to 312.50 µg/mL [70]. Ellagic acid previously isolated from *Miconia myriantha* [71] also showed moderate anti-*A. baumannii* activity, inhibiting 67% of bacterial growth at 250 µg/mL [72].

As a complementary investigation, three key compounds found in the chemical profile of the majority of samples **(**Table 4**)**, representing the main classes of secondary metabolites present in *M. albicans* extracts and phases –namely gallic acid (**3**), quercitrin (**10**), and corosolic acid (**18**)–, were selected and evaluated individually and in combination with ampicillin and ciprofloxacin against the bacterial strains (Table 1). For plant extracts and phases, the antibacterial activity is considered significant if MIC values are below 100 µg/mL, moderate if 100 ≤ MICs ≤ 625 µg/mL, and weak if MICs > 625 µg/mL [6, 40]. On the other hand, the antimicrobial activity of an isolated phytochemical has been defined as significant when MIC is below 10 µg/mL, moderate when 10 µg/mL < MIC < 100 µg/mL, and weak when MIC > 100 µg/mL [6, 73]. For *S. aureus* reference strain, compounds **3** and **18** showed moderate activity (MIC values of 31.3 and 62.5 µg/ml, respectively), while all the tested compounds proved ineffective against *E. coli*. Similarly, none of the compounds showed significant activities against clinical bacteria, with MIC values ranging from ≥ 500 to 250 µg/ml against MRSA and from ≥ 250 to 62.5 µg/ml against *A. baumannii*. When combined with antibiotics, these isolated compounds did not demonstrate any additive or synergistic effects (Tables 2 and 3). The scientific literature reports studies with analogous findings, where extracts and/or phases have shown superior activity compared to isolated compounds. Plant extracts and phases usually consist in complex mixtures of secondary metabolites, that can primarily act through multiple targets, exerting significant synergistic effects [6, 55, 74].

## Conclusions

Fighting microbial resistance requires a multifaceted approach that encompasses accurate diagnosis, appropriated prescription and adherence to treatment, proper disposal of antimicrobials, and significant investment in the development of new treatments. In this context, the search for new resistance-modifying agents finds in the vast biodiversity of plants an important source to be explored, in order to contribute to the development of new combinations that can act as adjuvants in the fight against resistant infections.

The extracts and phases obtained from *M. albicans* showed notable antibacterial properties, with specific emphasis on the activities of LEE extract and various phases, namely SCP, SHP, LEP e LHxP. Among these, SCP demonstrated the most effective antibiofilm activity and exerted synergistic effects when combined with ampicillin (AMP) against MRSA, and with ciprofloxacin (CIP) against *A. baumannii*. A synergic action against MRSA was also revealed by a combination of SHP with AMP, while synergistic interactions against the former microorganism were also observed for combinations of LEP with CIP and of LHxP with either AMP or CIP. In terms of the extracts, LEE showed the best activity profile against MRSA when combined with AMP. It is also worth to mention that extracts and phases from *M. albicans* showed higher antibacterial properties when compared to the isolated compounds gallic acid, quercitrin and corosolic acid, present in most of the samples.

The present findings provide evidence of the remarkable antimicrobial properties exhibited by *M. albicans*, particularly in its role as an adjuvant for antibiotic drugs, with potentialities for the development of novel efficacious agents aimed at treating and preventing the dissemination of antibiotic-resistant bacterial infections. Further comprehensive toxicological studies are required for safety purposes.

### Supplementary Information

**Additional file 1:** General experimental procedures. **Figure 1S.** - **Figure 38S.**

## Data Availability

All data generated or analysed during this study are included in this published article [and its supplementary information files].

## Abbreviations

LEE: Leaves-ethanol extract
LHxP: Leaves-hexane phase
LEP: Leaves-ethyl acetate phase
LCP: Leaves-chloroform phase
LHP: Leaves-hydromethanolic phase
SEE: Stems-ethanol extract
SHxP: Stems-hexane phase
SEP: Stems-ethyl acetate phase
SCP: Stems-chloroform phase
SHP: Stems-hydromethanolic phase
HPLC–DAD-MS/MS: High-Performance Liquid Chromatography coupled with Diode Array Detector and tandem Mass Spectrometry
ESI: Electrospray ionization
GC–MS: Gas Chromatography Coupled to Mass Spectrometry
NMR: Nuclear Magnetic Resonance
MDR: Multi-drug resistant
MRSA: Methicillin-resistant *Staphylococcus aureus*
CDCl_3_: Deuterated chloroform
CD_3_OD: Deuterated methanol
EtOAc: Ethyl acetate
MeOH: Methanol
RR: Relative retention
CFU: Colony-forming unit
TTC: Triphenyltetrazolium chloride
MIC: Minimum inhibitory concentration
FIC: Fractional Inhibitory Concentration
FICI: Fractional Inhibitory Concentration index
DMSO: Dimethyl sulfoxide
EtOH: Ethanol
UV: Ultraviolet
HRESIMS: High Resolution Electrospray Ionization Mass Spectrometry
MS/MS: Tandem Mass Spectrometry
MSSA: Methicillin-Sensitive *S. aureus*
DCM: Dichloromethane
