# Supplementary material for: Medicinal plant Miconia albicans synergizes with ampicillin and ciprofloxacin against multi-drug resistant Acinetobacter baumannii and Staphylococcus aureus
Source: BMC Complement Med Ther. 2023 Oct 23;23:374. doi: 10.1186/s12906-023-04147-w (PMC10594757; doi:10.1186/s12906-023-04147-w)

SUPPLEMENTARY MATERIAL

Medicinal plant *Miconia albicans* synergizes with ampicillin and ciprofloxacin against multi-drug resistant *Acinetobacter baumannii* and *Staphylococcus aureus*

Genilson Silva de Jesus^1^, Danielle da Silva Trentin^2^, Thayná Fernandes Barros^2^, Alda Maria Teixeira Ferreira^3^, Bruna Castro de Barros^3^, Patrícia de Oliveira Figueiredo^1^, Fernanda Rodrigues Garcez^1^, Érica Luiz dos Santos^1^, Ana Camila Micheletti^1^*, Nidia Cristiane Yoshida^1^*

^1^Laboratório de Produtos Naturais Bioativos-PRONABio, Instituto de Química, Universidade Federal de Mato Grosso do Sul, Campo Grande, Brazil

^2^ Laboratório de Bacteriologia & Modelos Experimentais Alternativos (BACMEA), Departamento de Ciências Básicas da Saúde, Universidade Federal de Ciências da Saúde de Porto Alegre, Porto Alegre, Brazil

^3^ Instituto de Biociências, Universidade Federal de Mato Grosso do Sul, Campo Grande, Brazil

Corresponding Author: Nidia Cristiane Yoshida and Ana Camila Micheletti
nidia.yoshida@ufms.br and anamicheletti@gmail.com

**General Experimental Procedures**

HRESIMS data were acquired with electrospray ionization in negative and positive ion mode on an UltrOTOF-Q instrument (Bruker Daltonics, Billerica, MA, USA). NMR spectroscopic data were recorded at room temperature in CDCl_3_ and CD_3_OD (Cambridge Isotope Laboratories, Andover, MA, USA) on a Bruker DPX-300 spectrometer (Bruker, Karlhue, Germany) operating at 300.13 MHz (^1^H)/75.47 MHz (^13^C).

**Figure 1S.** MS data of compound 1

**Figure 2S.** MS data of compound 2

**Figure 3S.** MS data of compound 3

**Figure 4S.** MS data of compound 4

**Figure 5S.** MS data of compound 5

**Figure 6S.** MS data of compound 6

**Figure 7S.** MS data of compound 7

**Figure 8S.** MS data of compound 8

**Figure 9S.** MS data of compound 9

**Figure 10S.** MS data of compound 10

**Figure 11S.** MS data of compound 11

**Figure 12S.** MS data of compound 12

**Figure 13S.** MS data of compound 13

**Figure 14S.** MS data of compound 14

**Figure 15S.** MS data of compound 15

**Figure 16S.** MS data of compound 16

**Figure 17S.** MS data of compound 17

**Figure 18S.** MS data of compound 18

**Figure 19S.** MS data of compound 19

**Figure 20S.** MS data of compound 20

**Figure 21S**. MS data of compound 21

**Figure 22S**. ^1^H NMR spectrum (300 MHz, CD_3_OD) of compound 10 (quercitrin)

**Figure 23S**. ^13^C NMR spectrum (75 MHz, CD_3_OD) of compound 10 (quercitrin)

**Figure 24S**. ^1^H NMR spectrum (300 MHz, CD_3_OD) of compound 18 (corosolic acid)

**Figure 25S**. ^13^C NMR spectrum (75 MHz, CD_3_OD) of compound 18 (corosolic acid)

**Figure 26S**. ^1^H NMR spectrum (300 MHz, CDCl_3_) of compound 19 (betulinic acid)

**Figure 27S**. ^13^C NMR spectrum (75 MHz, CDCl_3_) of compound 19 (betulinic acid)

**Figure 28S**. ^1^H NMR spectrum (300 MHz, CDCl_3_) of compound 20 (Pheophorbide B)

**Figure 29S**. ^13^C NMR spectrum (75 MHz, CDCl_3_) of compound 20 (Pheophorbide B)

**Figure 30S**. ^1^H NMR spectrum (300 MHz, CDCl_3_) of compound 21 (Pheophorbide A ethyl ester)

**Figure 31S**. ^13^C NMR spectrum (75MHz, CDCl_3_) of compound 21 ((Pheophorbide A ethyl ester)

**Figure 32S**. ^1^H NMR spectrum (300 MHz, CDCl_3_) of a mixture of compound 23 and 25

**Figure 33S**. ^13^C NMR spectrum (75 MHz, CDCl_3_) of a mixture of compound 23 (squalene) and 25 (α-tocopherol)

**Figure 34S**. ^1^H NMR spectrum (300 MHz, CDCl_3_) of compound 28 (β-sitosterol)

**Figure 35S**. ^13^C NMR spectrum (75MHz, CDCl_3_) of compound 28 (β-sitosterol)

**Figure 36S**. ^1^H NMR spectrum (300 MHz, CDCl_3_) of a mixture of compound 29 and 31

**Figure 37S**. ^13^C NMR spectrum (75 MHz, CDCl_3_) of a mixture of compound 29 (β-amyrin) and 31 (α-amyrin)

**Figure 38S.** HPLC-DAD-MS/MS (positive mode) profiles of ethanol extracts and phases of *M. albicans*, highlighting the presence of pheophorbides **20** and **21**.

Peak numbers refer to compounds listed in Table 4. LEE = Leaves-ethanol extract. LCP= Leaves-chloroform phase. LEP= Leaves-ethyl acetate phase. LHP= Leaves-hydromethanolic phase. SEE = Stems-ethanol extract. SCP= Stems-chloroform phase. SEP= Stems-ethyl acetate phase. SHP= Stems-hydromethanolic phase.

**Figure 1S**. MS data of compound 1


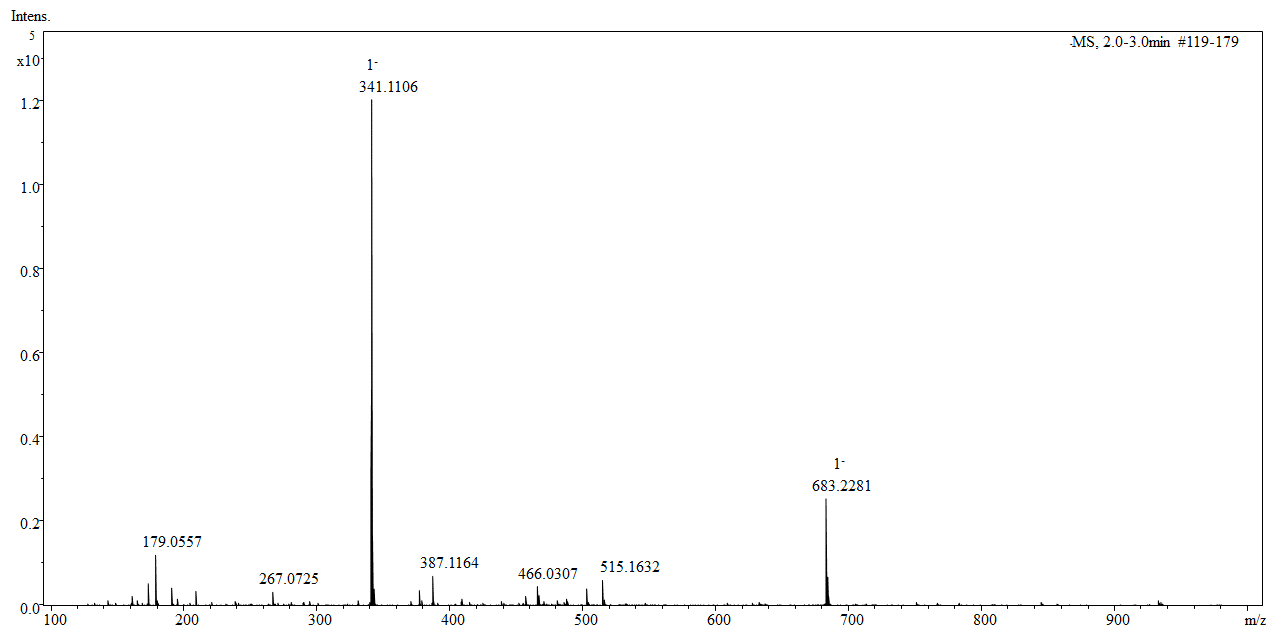


**Figure 2S**. MS data of compound 2


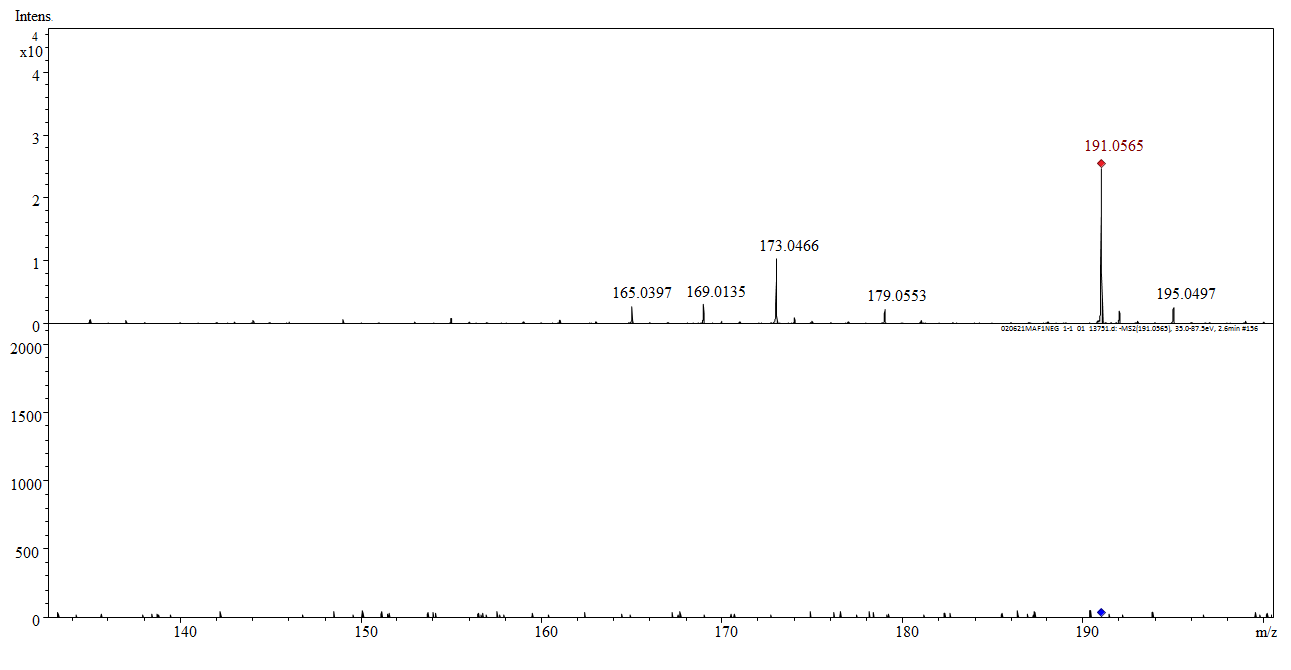


**Figure 3S**. MS data of compound 3


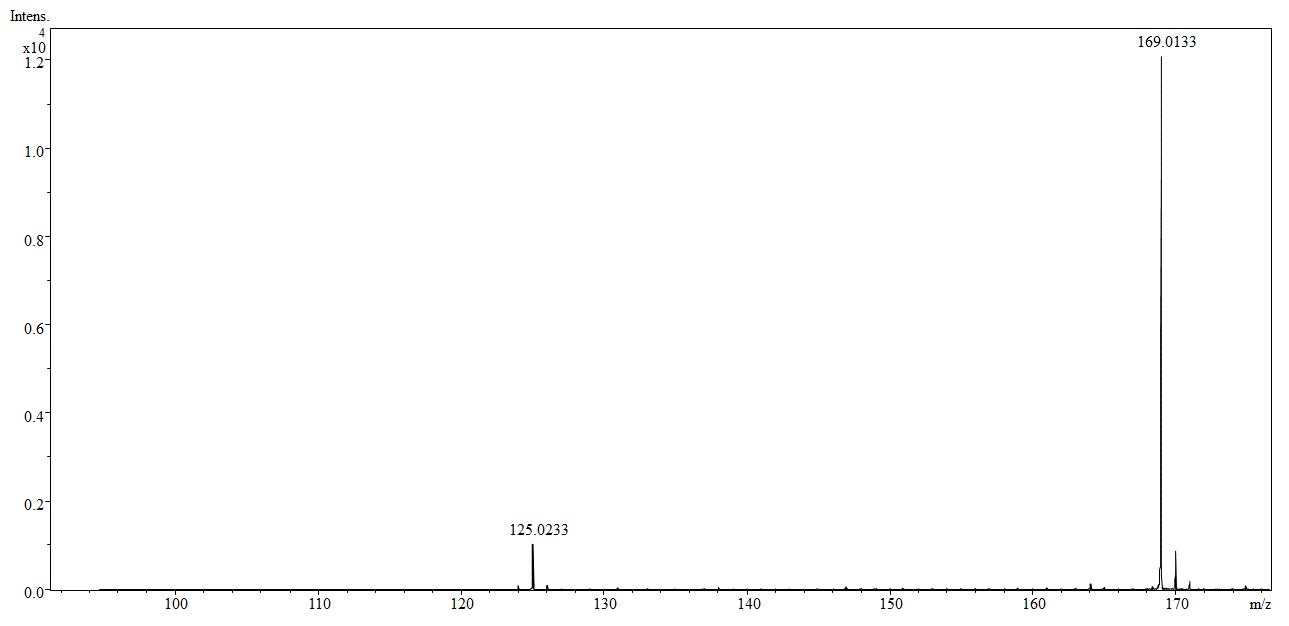


**Figure 4S**. MS data of compound 4


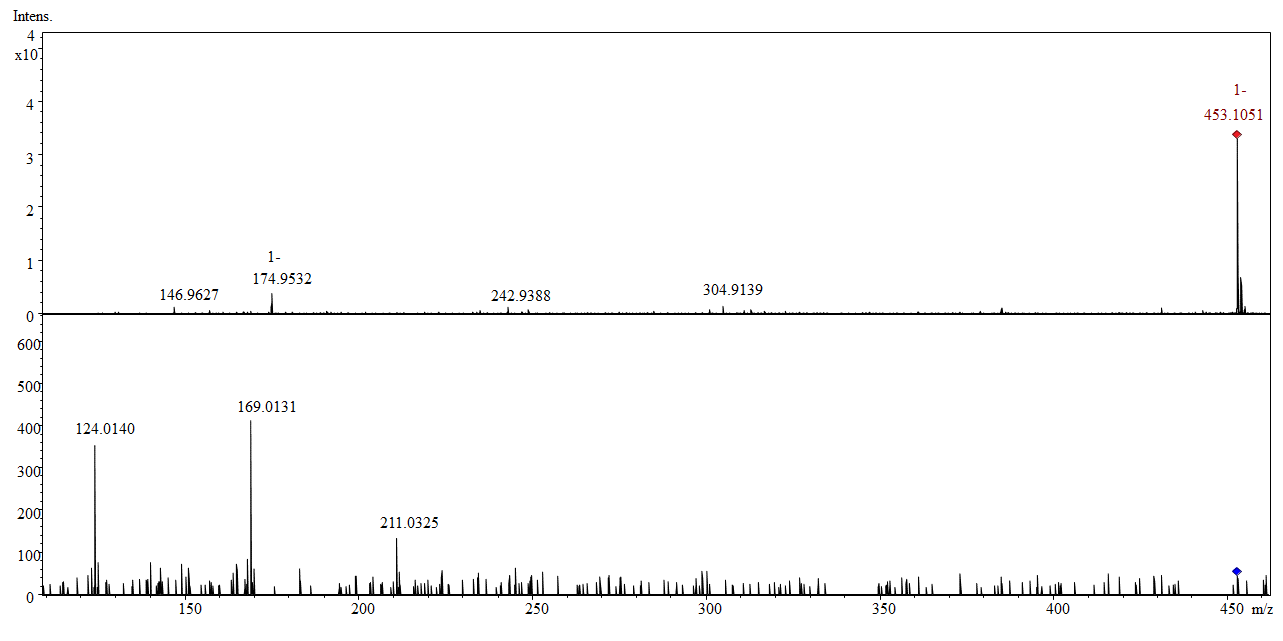


**Figure 5S**. MS data of compound 5


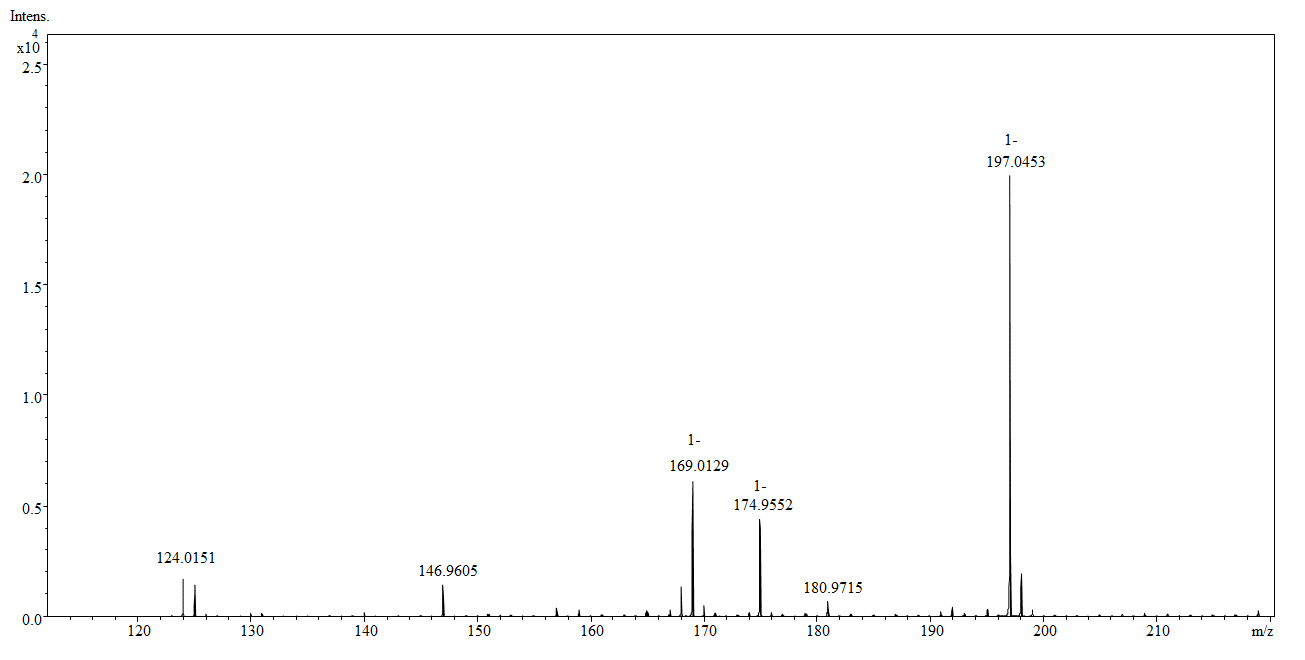


**Figure 6S**. MS data of compound 6


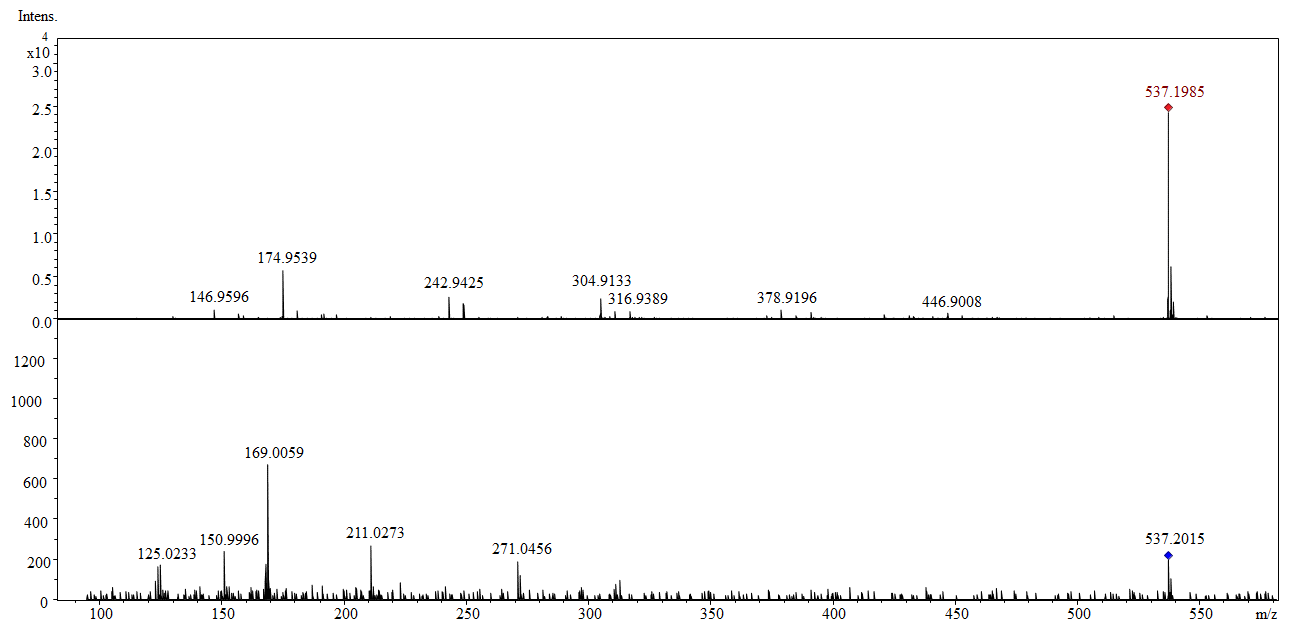


**Figure 7S**. MS data of compound 7


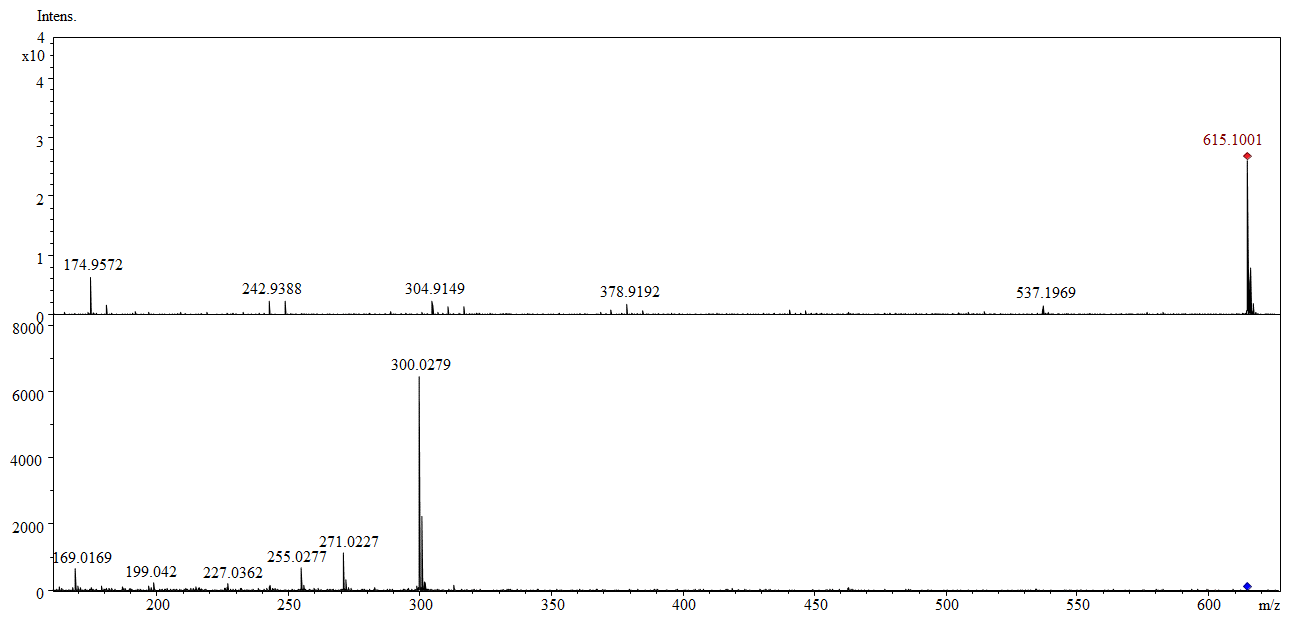


**Figure 8S**. MS data of compound 8


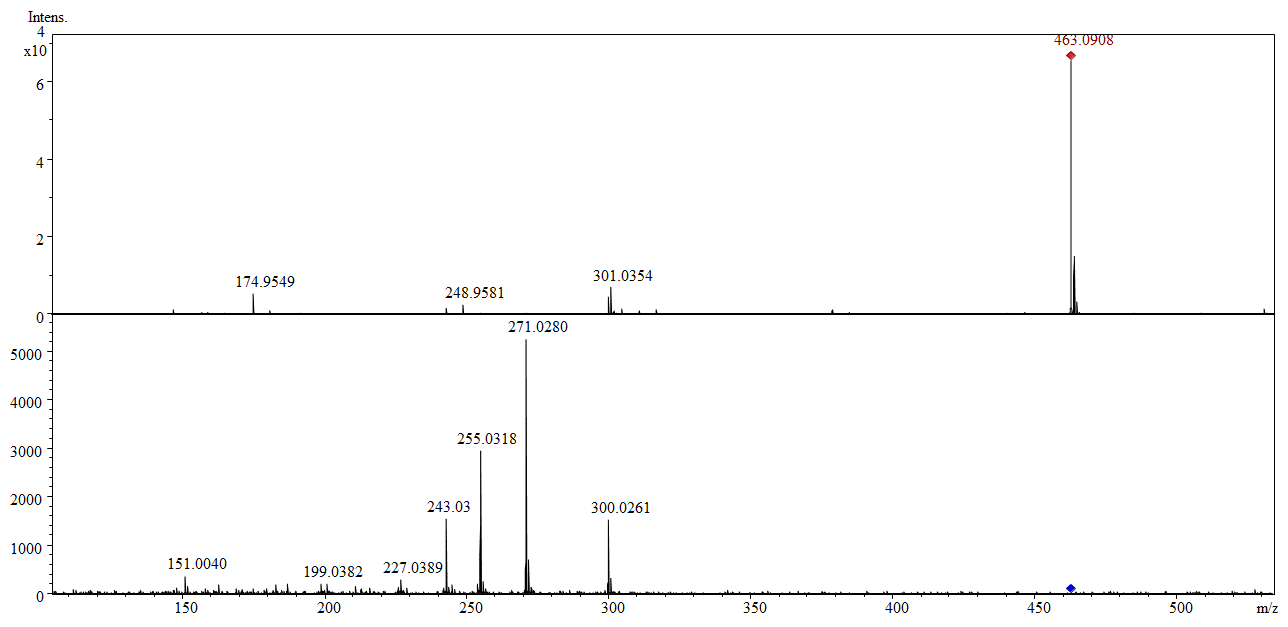


**Figure 9S**. MS data of compound 9


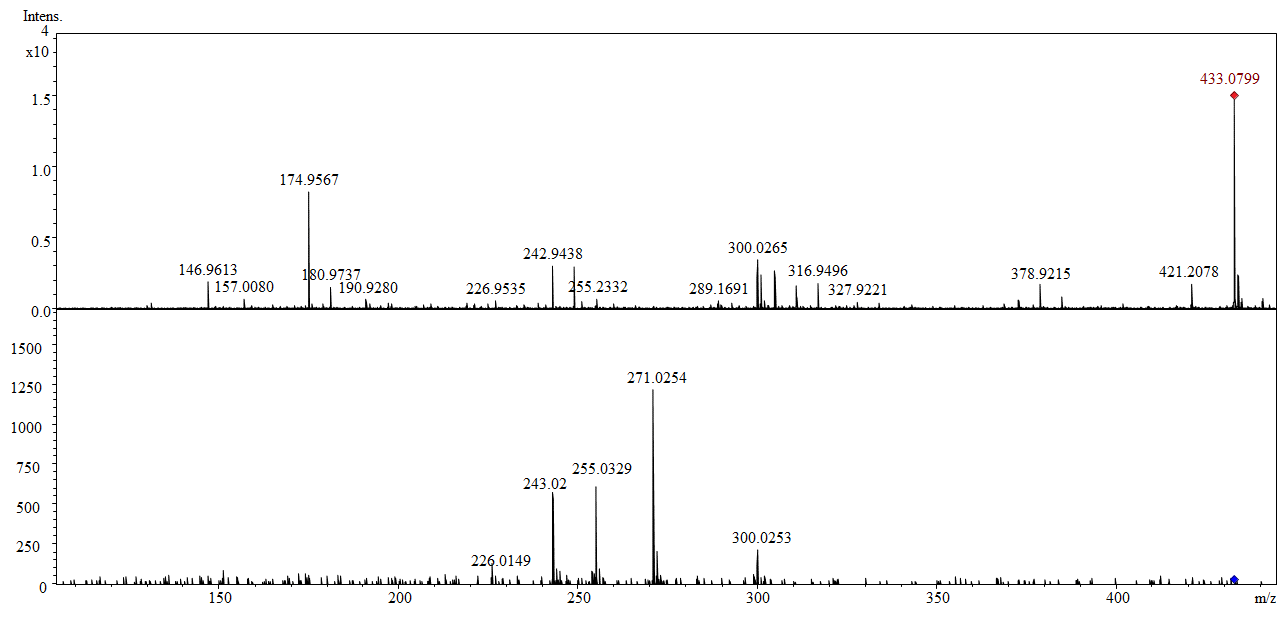


**Figure 10S**. MS data of compound 10


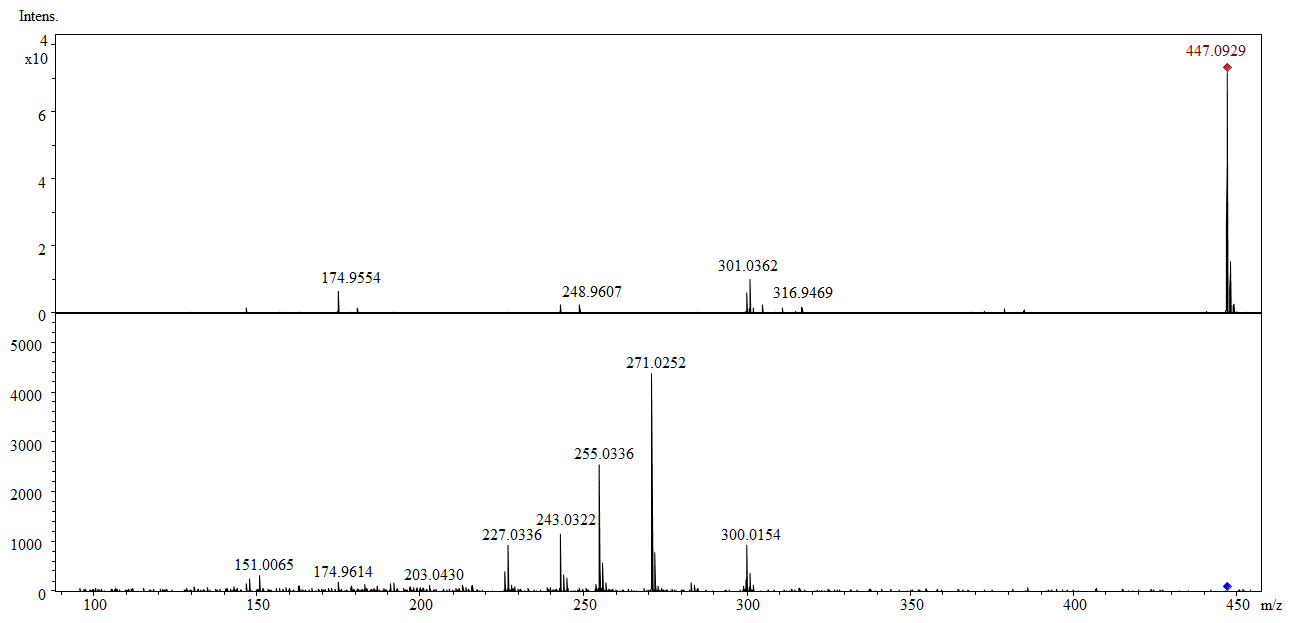


**Figure 11S**. MS data of compound 11


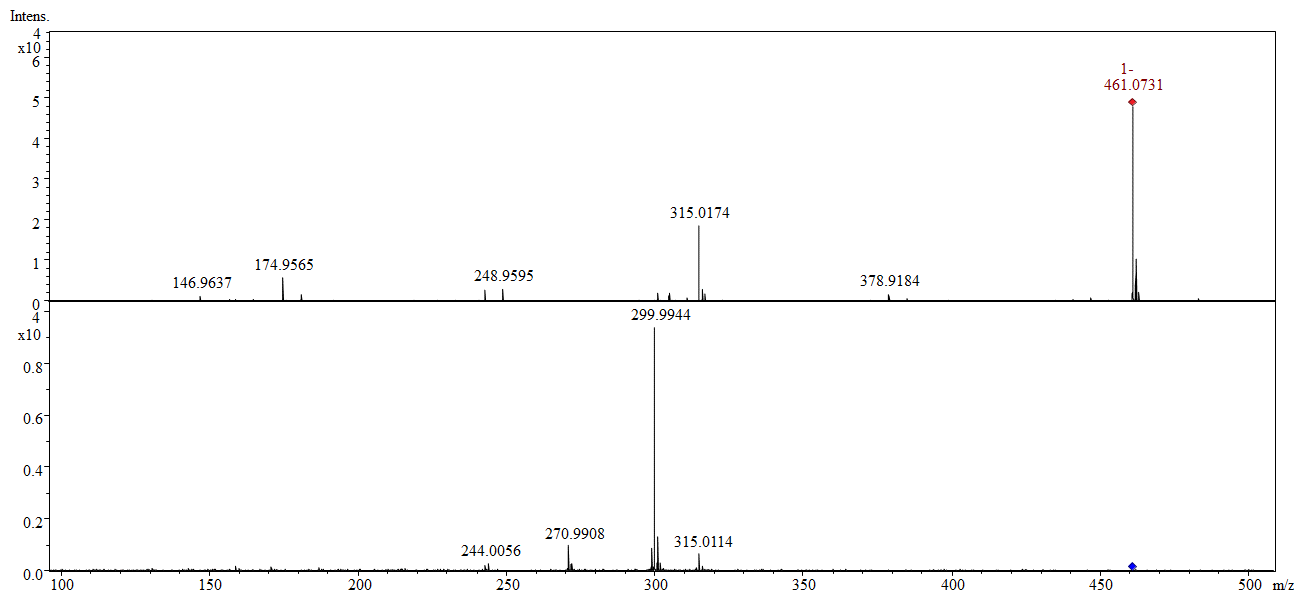


**Figure 12S**. MS data of compound 12


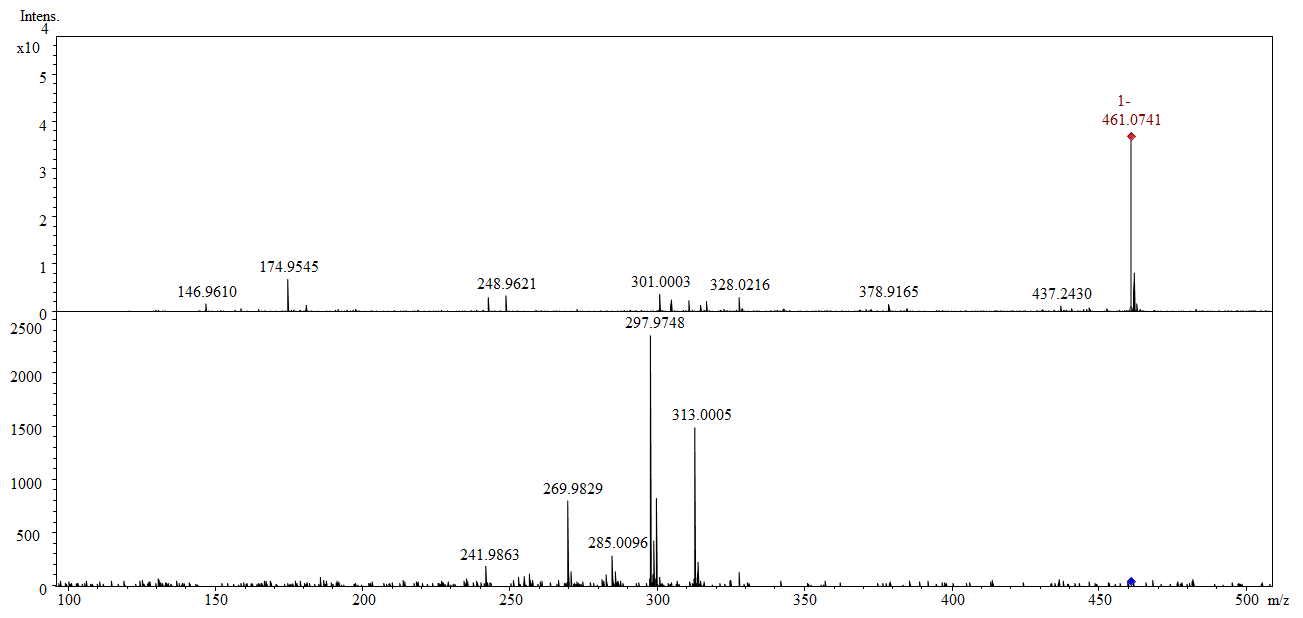


**Figure 13S**. MS data of compound 13


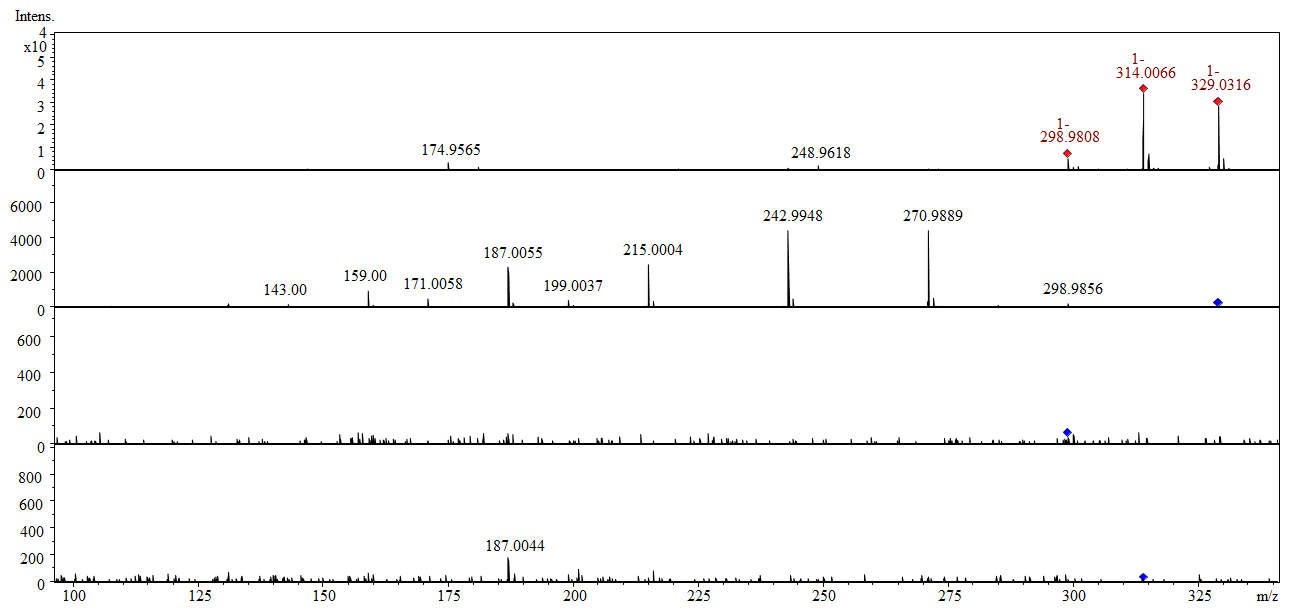


**Figure 14S**. MS data of compound 14


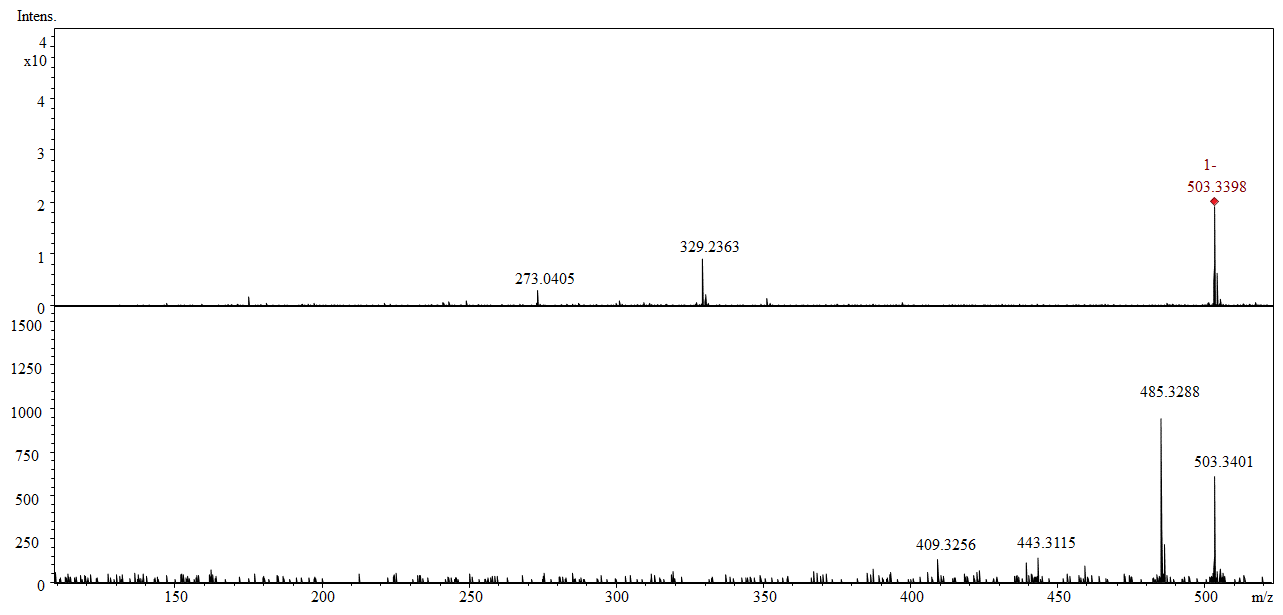


**Figure 15S**. MS data of compound 15


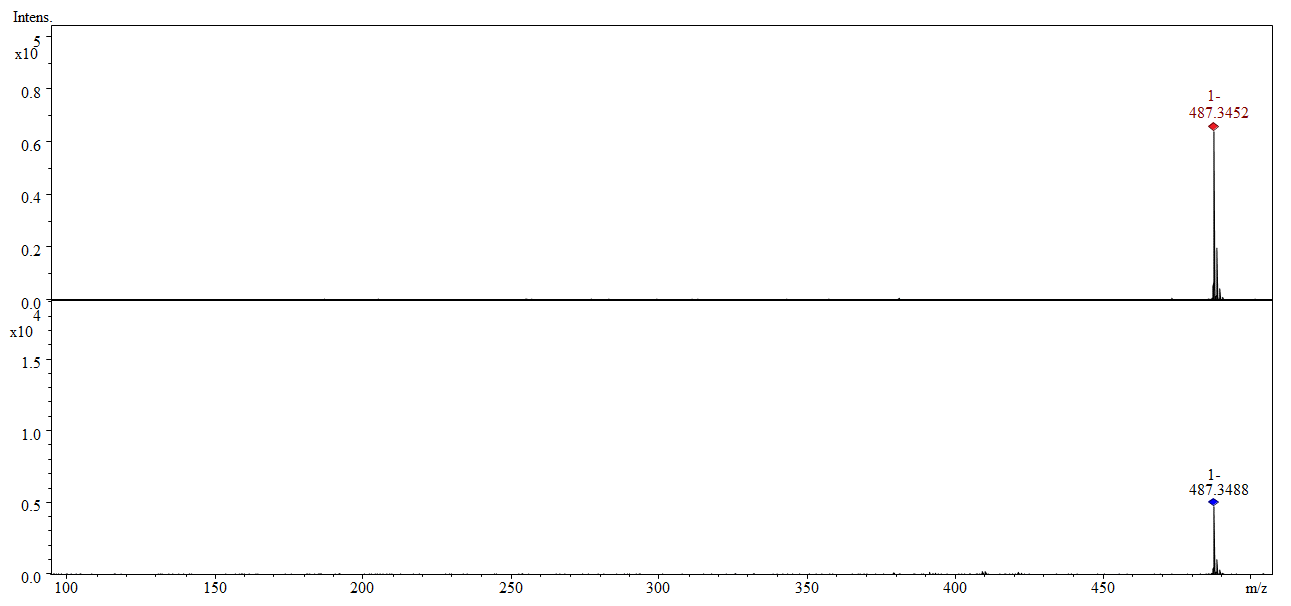


**Figure 16S**. MS data of compound 16


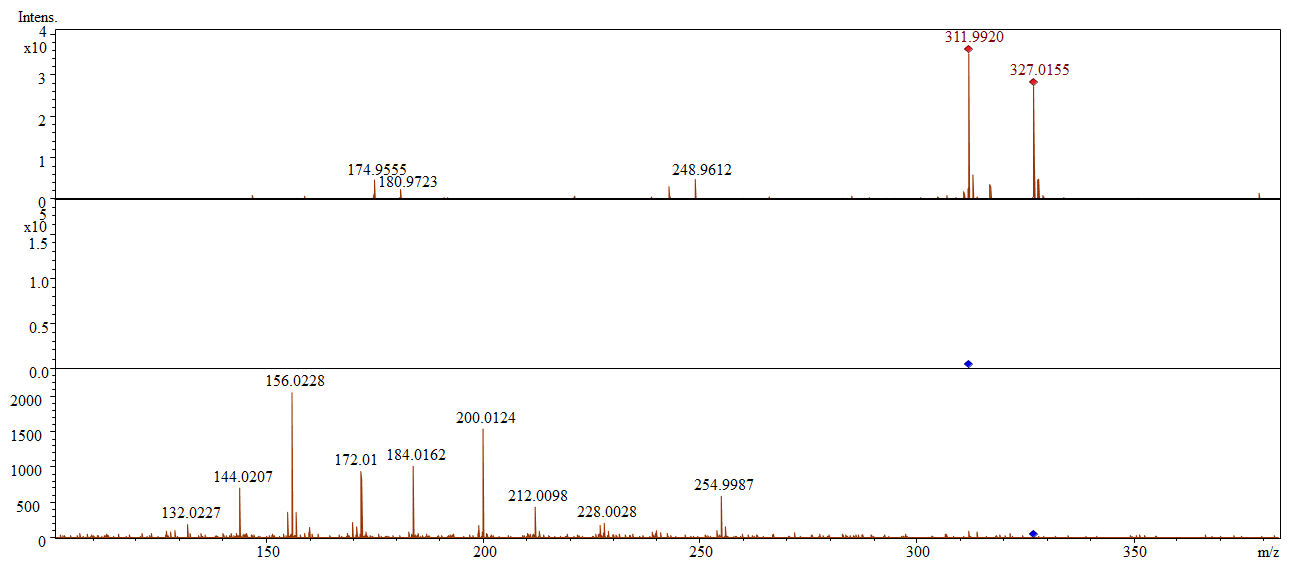


**Figure 17S**. MS data of compound 17


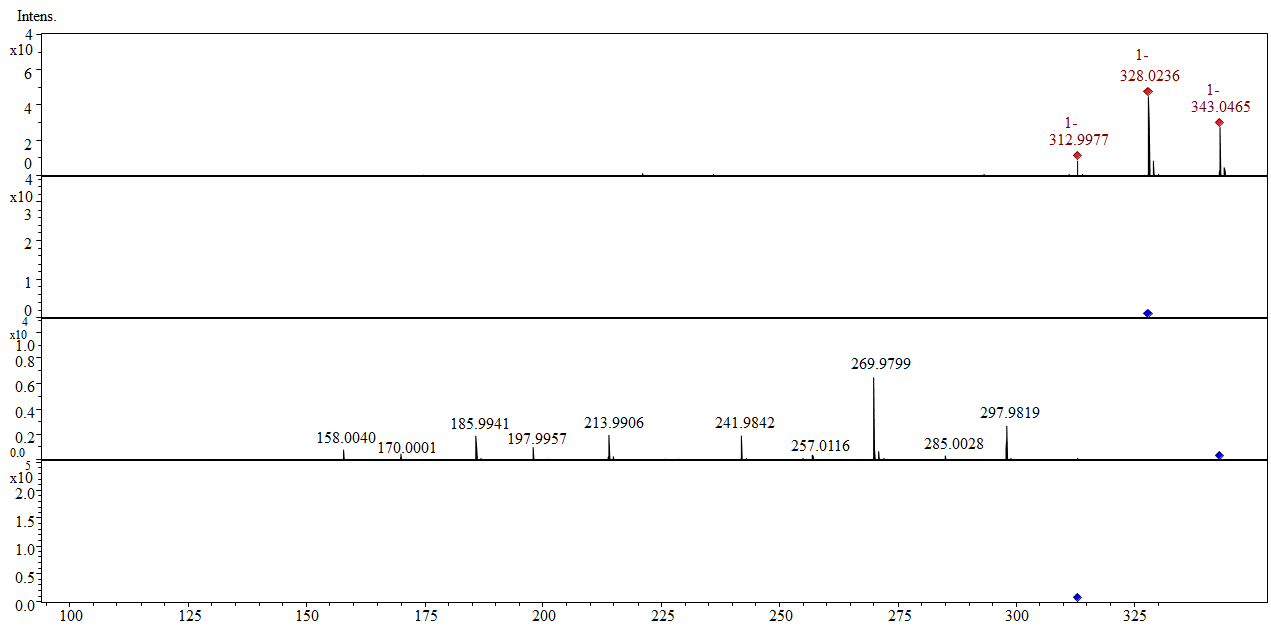


**Figure 18S**. MS data of compound 18


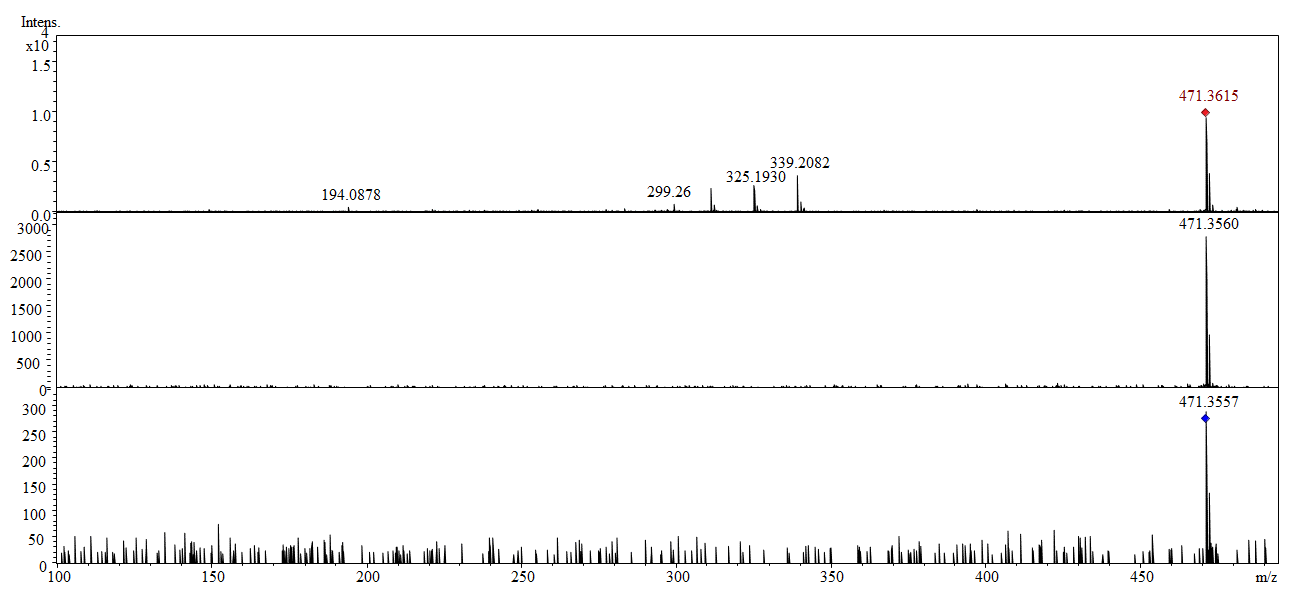


**Figure 19S**. MS data of compound 19


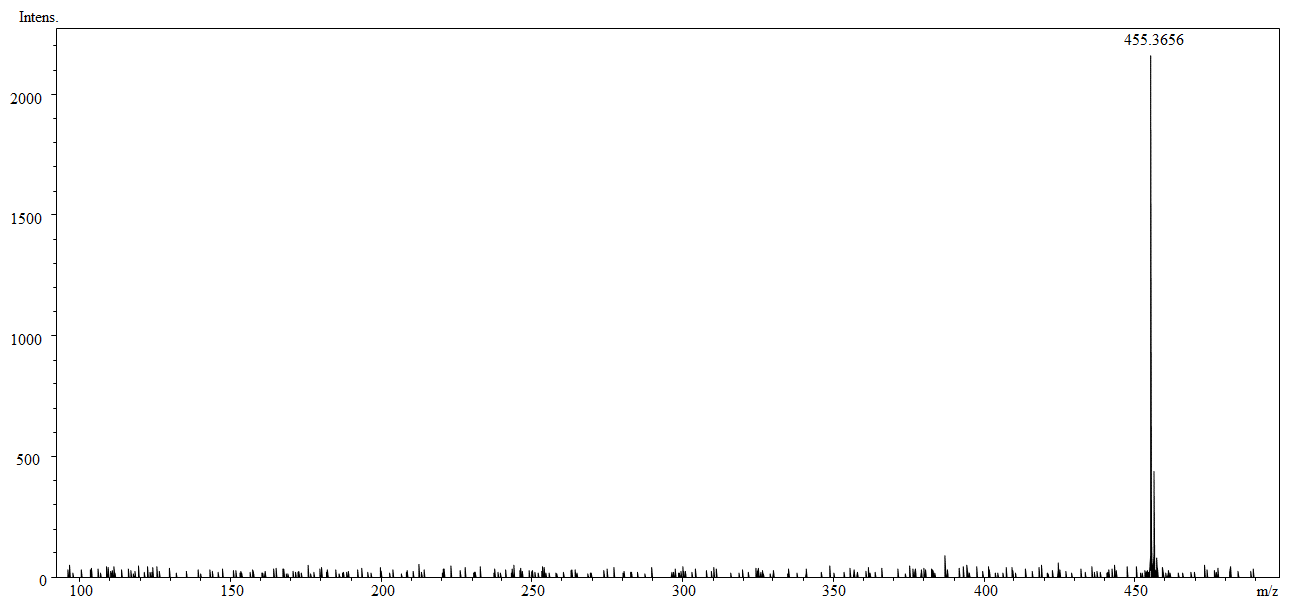


**Figure 20S**. MS data of compound 20


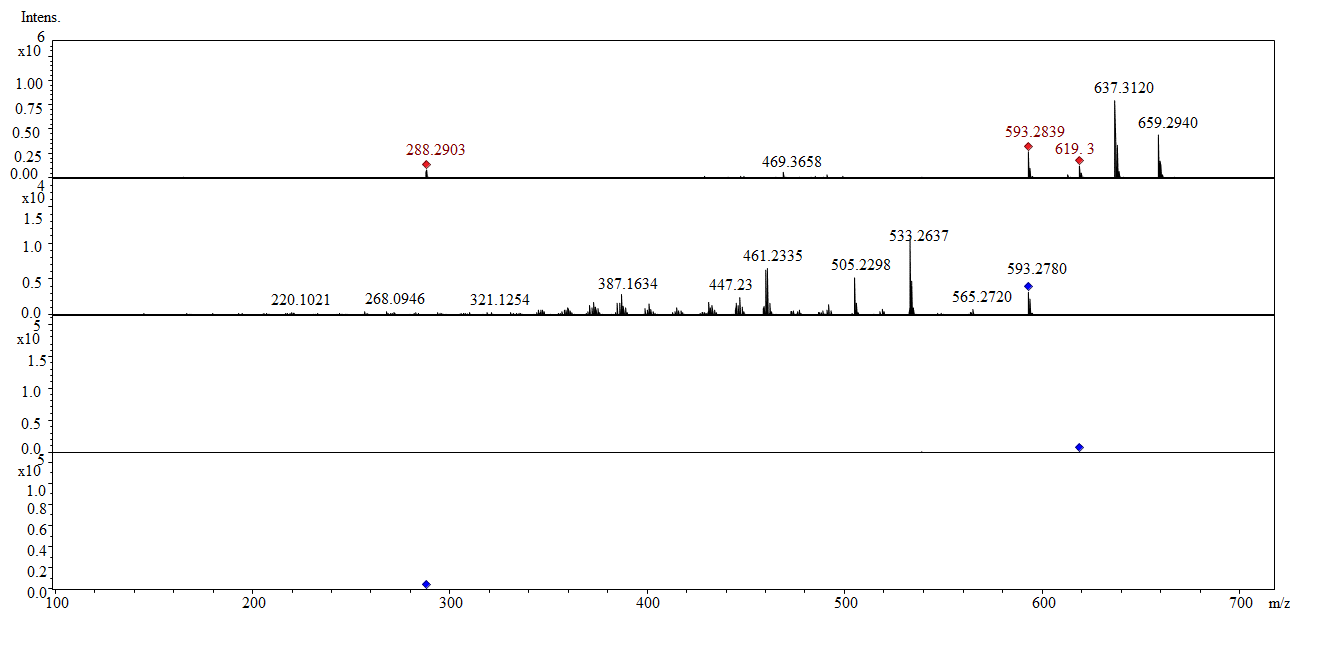


**Figure 21S**. MS data of compound 21


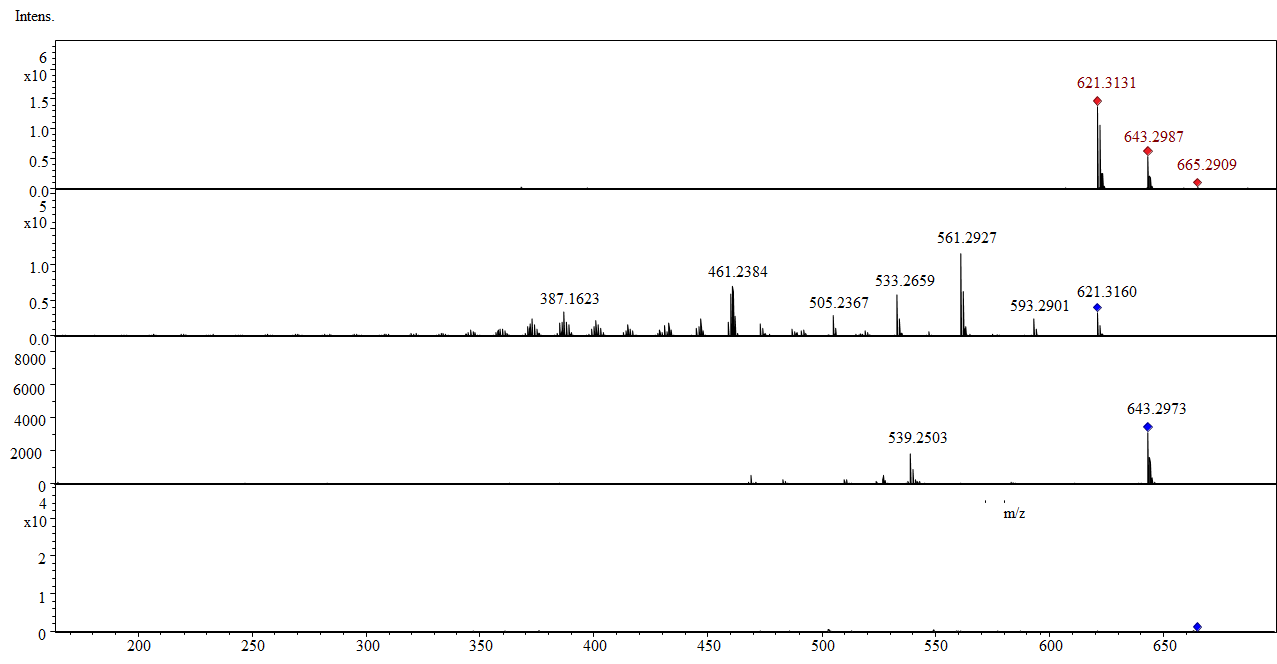


**Figure 22S**. ^1^H NMR spectrum (300 MHz, CD_3_OD) of compound 10 (quercitrin)


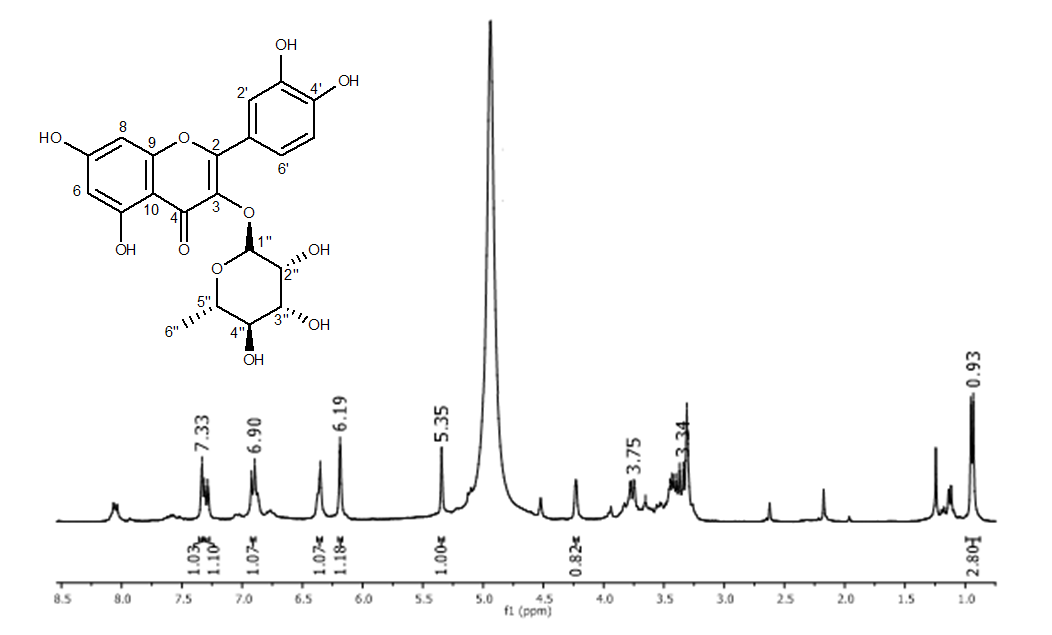


**Figure 23S**. ^13^C NMR spectrum (75 MHz, CD_3_OD) of compound 10 (quercitrin)

**Figure 24S**. ^1^H NMR spectrum (300 MHz, CD_3_OD) of compound 18 (corosolic acid)


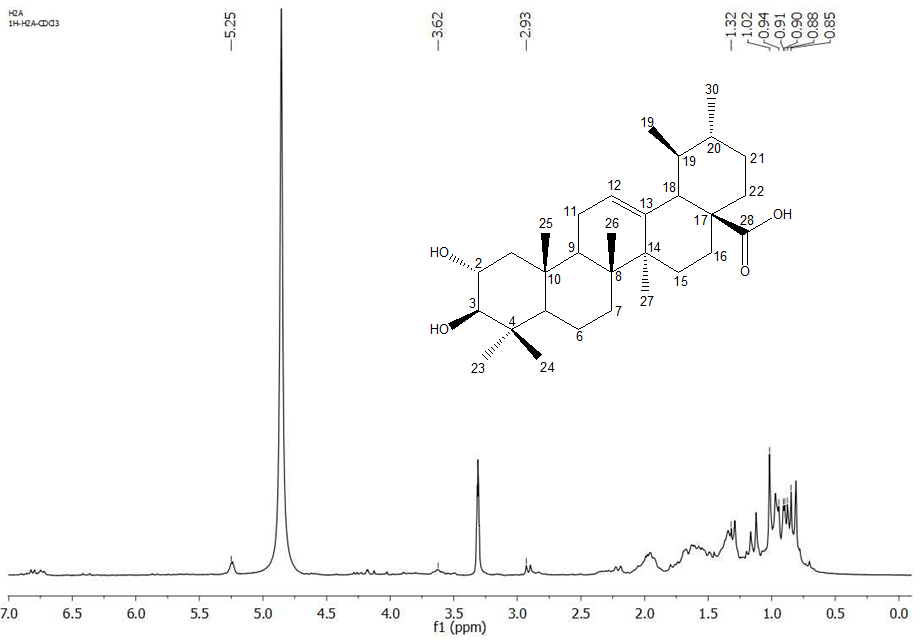


**Figure 25S**. ^13^C NMR spectrum (75 MHz, CD_3_OD) of compound 18 (corosolic acid)


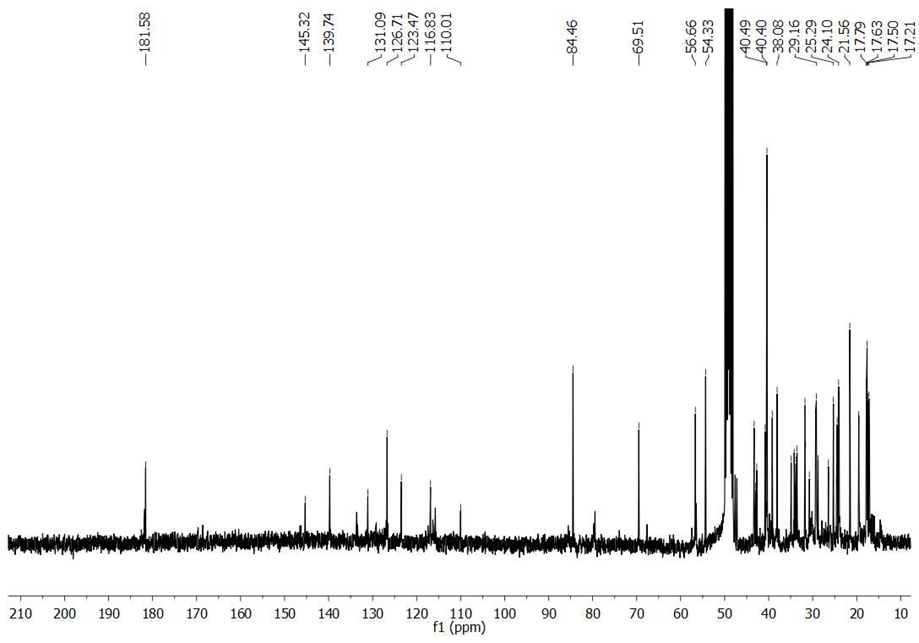


**Figure 26S**. ^1^H NMR spectrum (300 MHz, CDCl_3_) of compound 19 (betulinic acid)


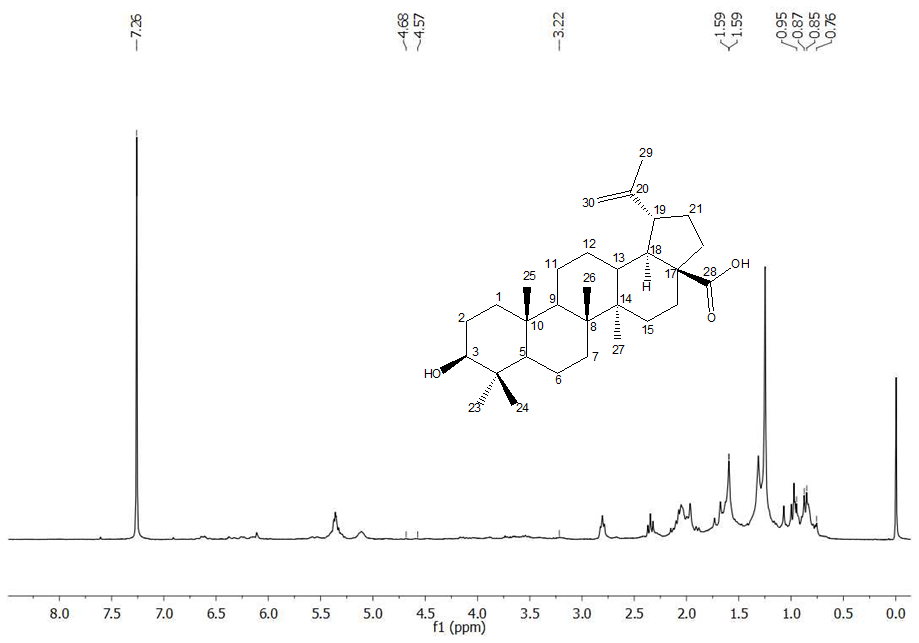


**Figure 27S**. ^13^C NMR spectrum (75 MHz, CDCl_3_) of compound 19 (betulinic acid)


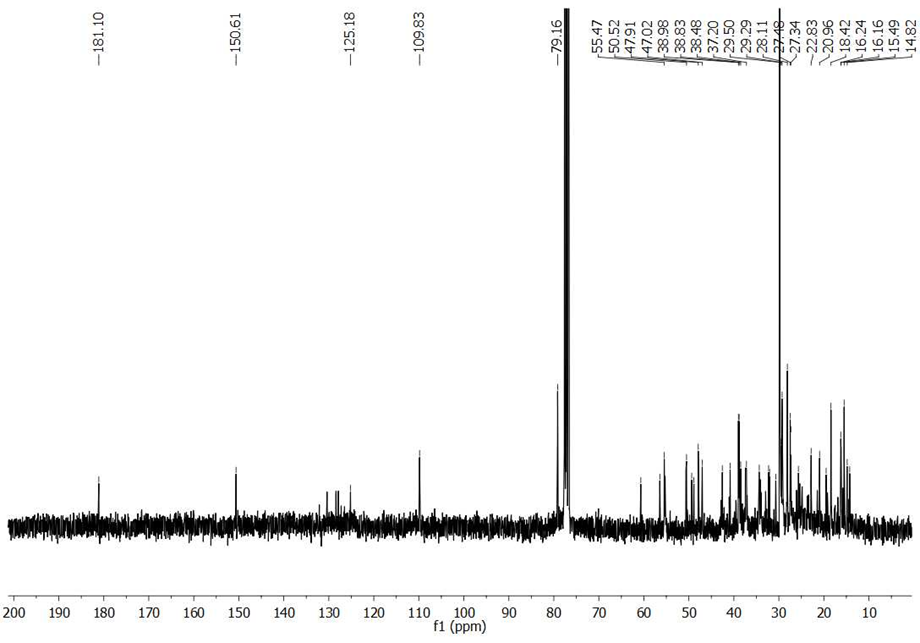


**Figure 28S**. ^1^H NMR spectrum (300 MHz, CDCl_3_) of compound 20 (Pheophorbide B)


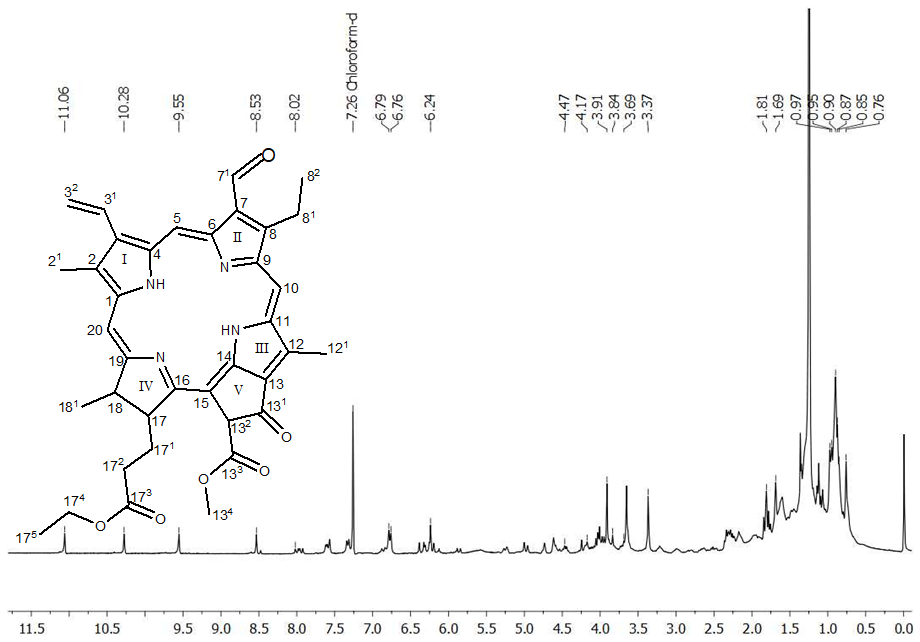


**Figure 29S**. ^13^C NMR spectrum (75 MHz, CDCl_3_) of compound 20 (Pheophorbide B)


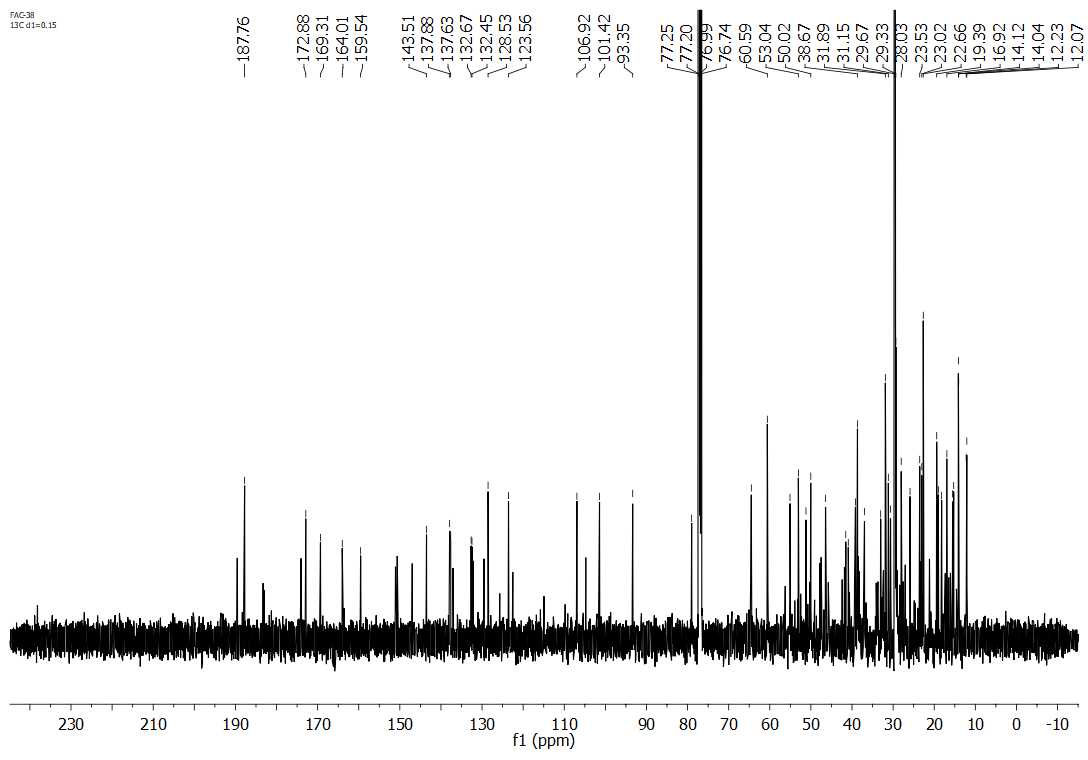


**Figure 30S**. ^1^H NMR spectrum (300 MHz, CDCl_3_) of compound 21 (Pheophorbide A ethyl ester)


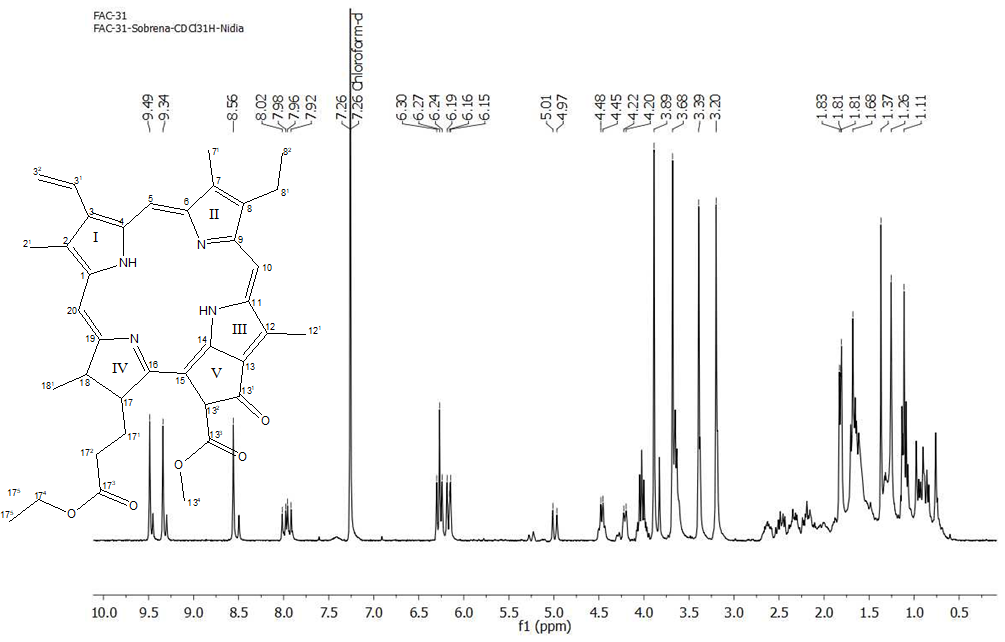


**Figure 31S**. ^13^C NMR spectrum (75MHz, CDCl_3_) of compound 21 ((Pheophorbide A ethyl ester)


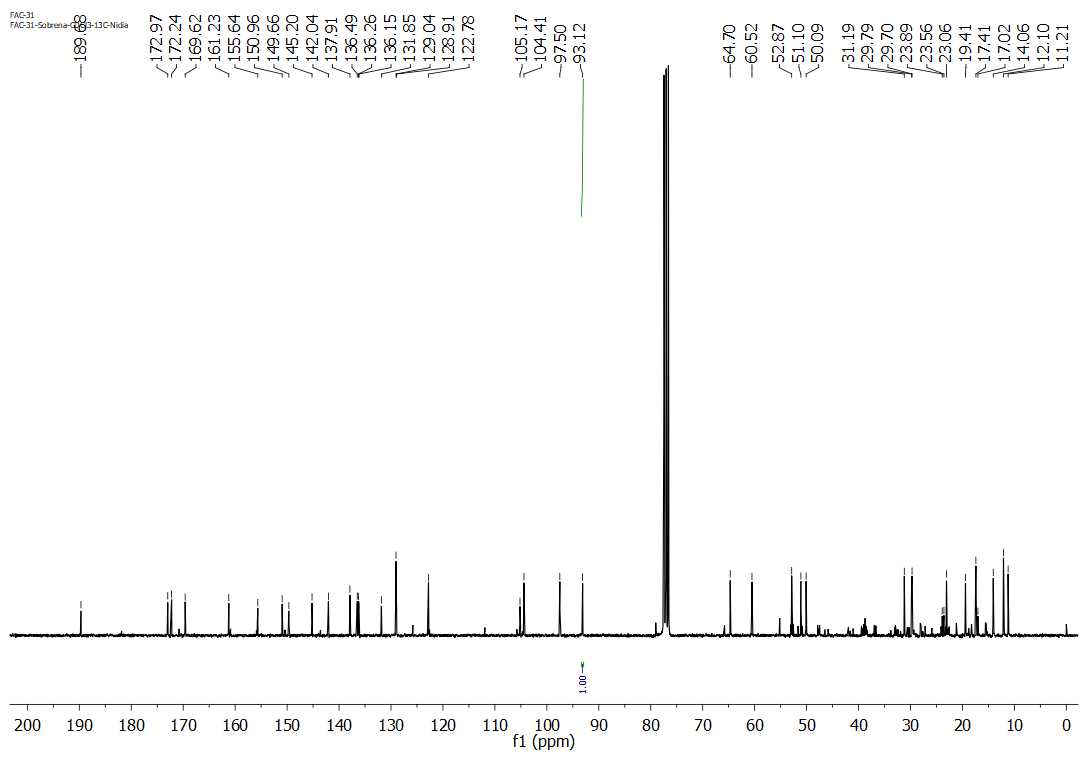


**Figure 32S**. ^1^H NMR spectrum (300 MHz, CDCl_3_) of a mixture of compound 23 and 25


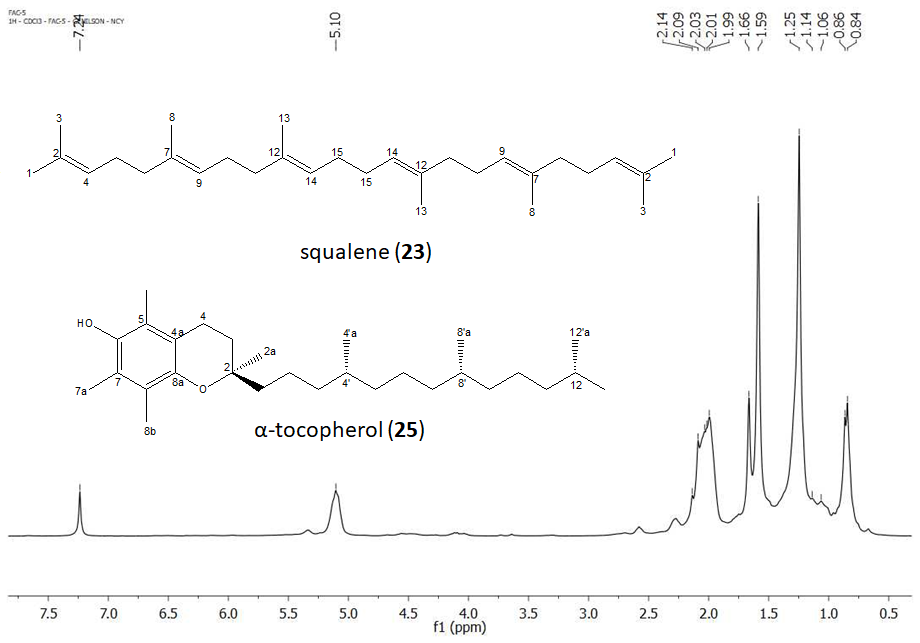


**Figure 33S**. ^13^C NMR spectrum (75 MHz, CDCl_3_) of a mixture of compound 23 (squalene) and 25 (α-tocopherol)


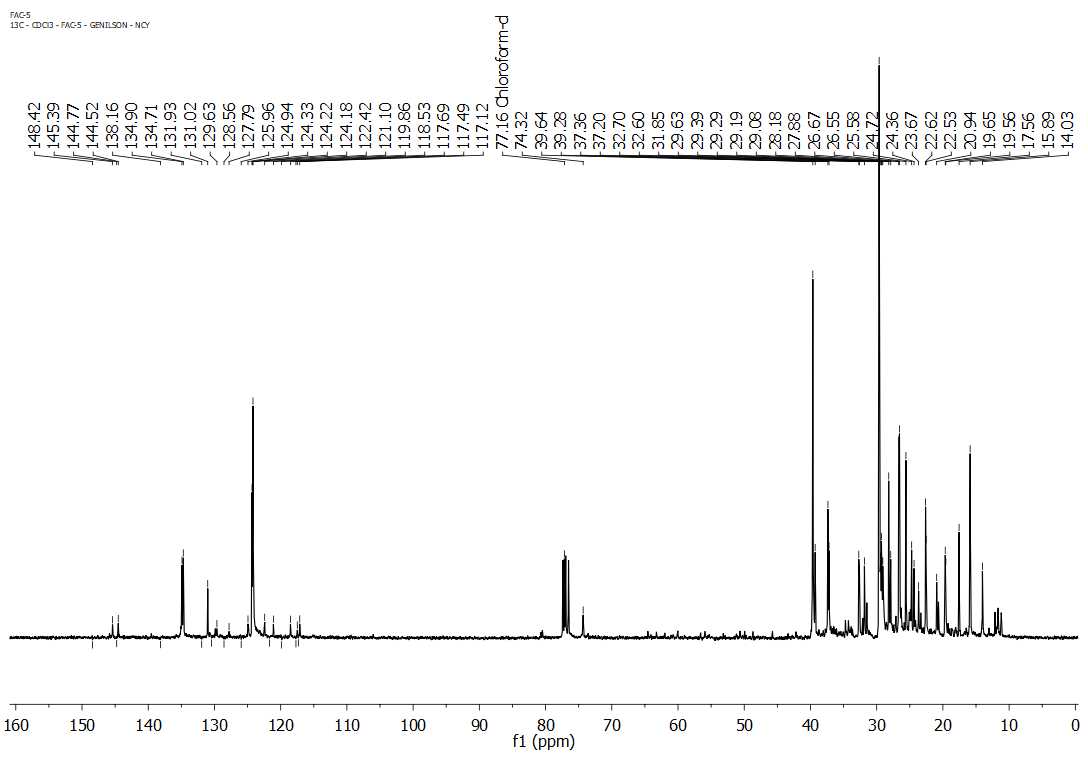


**Figure 34S**. ^1^H NMR spectrum (300 MHz, CDCl_3_) of compound 28 (β-sitosterol)


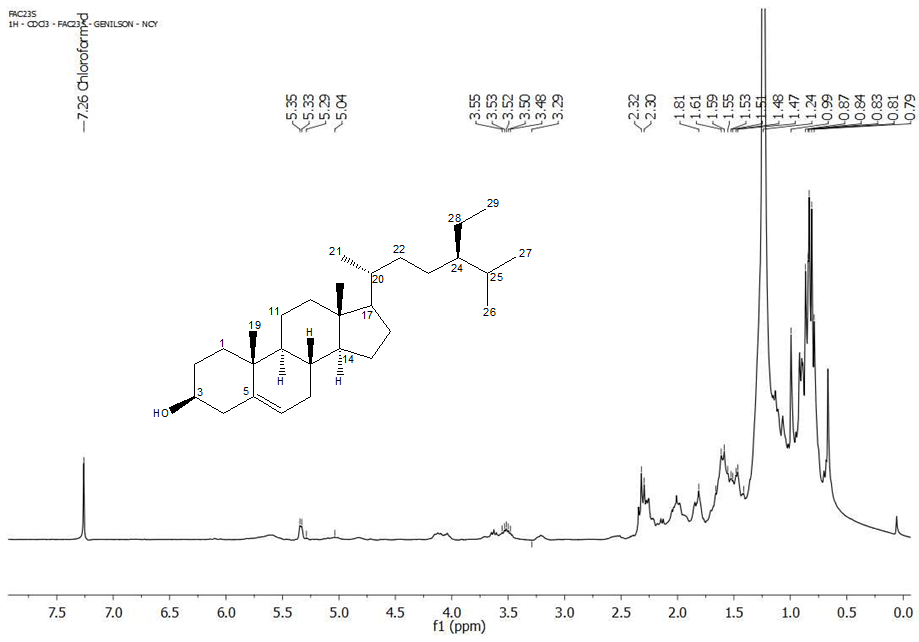


**Figure 35S**. ^13^C NMR spectrum (75MHz, CDCl_3_) of compound 28 (β-sitosterol)


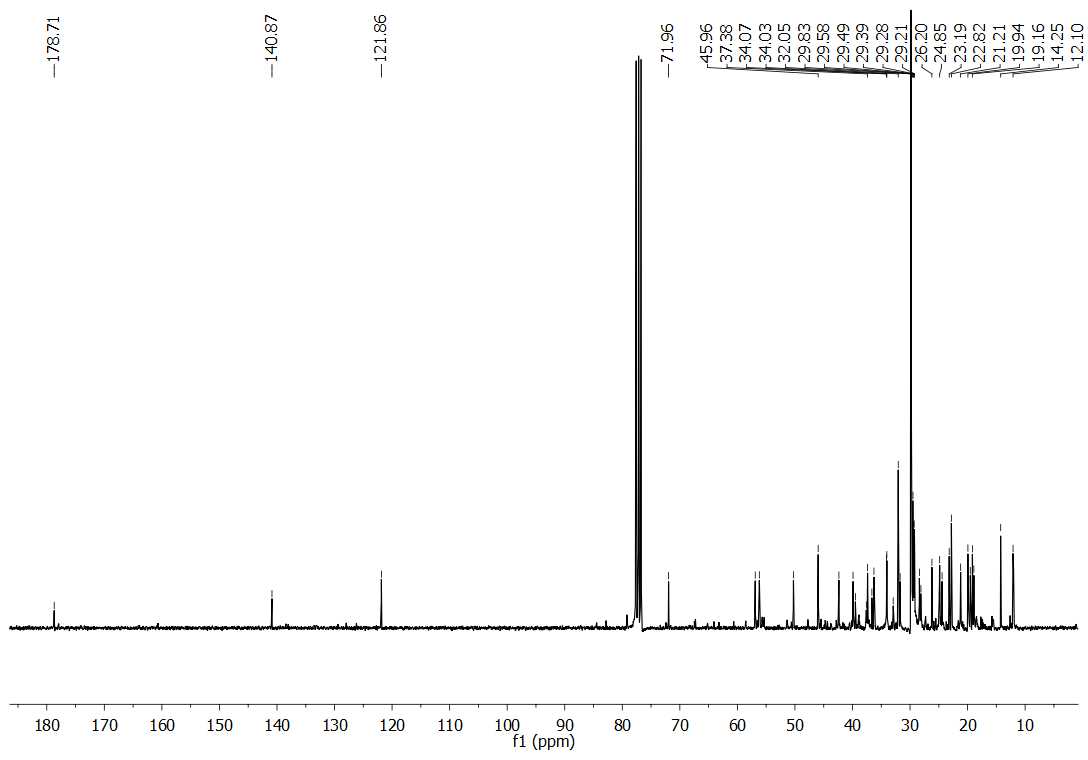


**Figure 36S**. ^1^H NMR spectrum (300 MHz, CDCl_3_) of a mixture of compound 29 and 31


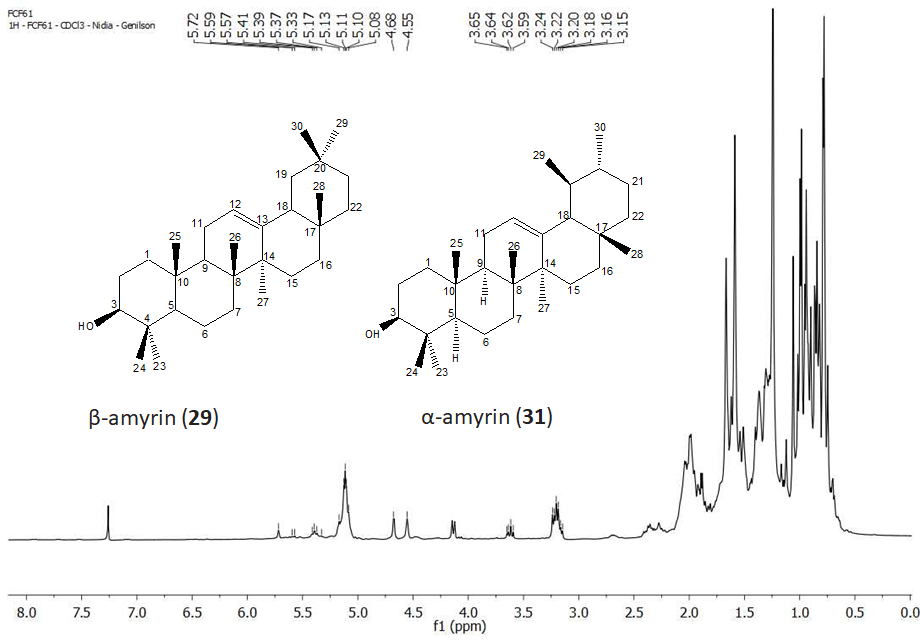


**Figure 37S**. ^13^C NMR spectrum (75 MHz, CDCl_3_) of a mixture of compound 29 (β-amyrin) and 31 (α-amyrin)


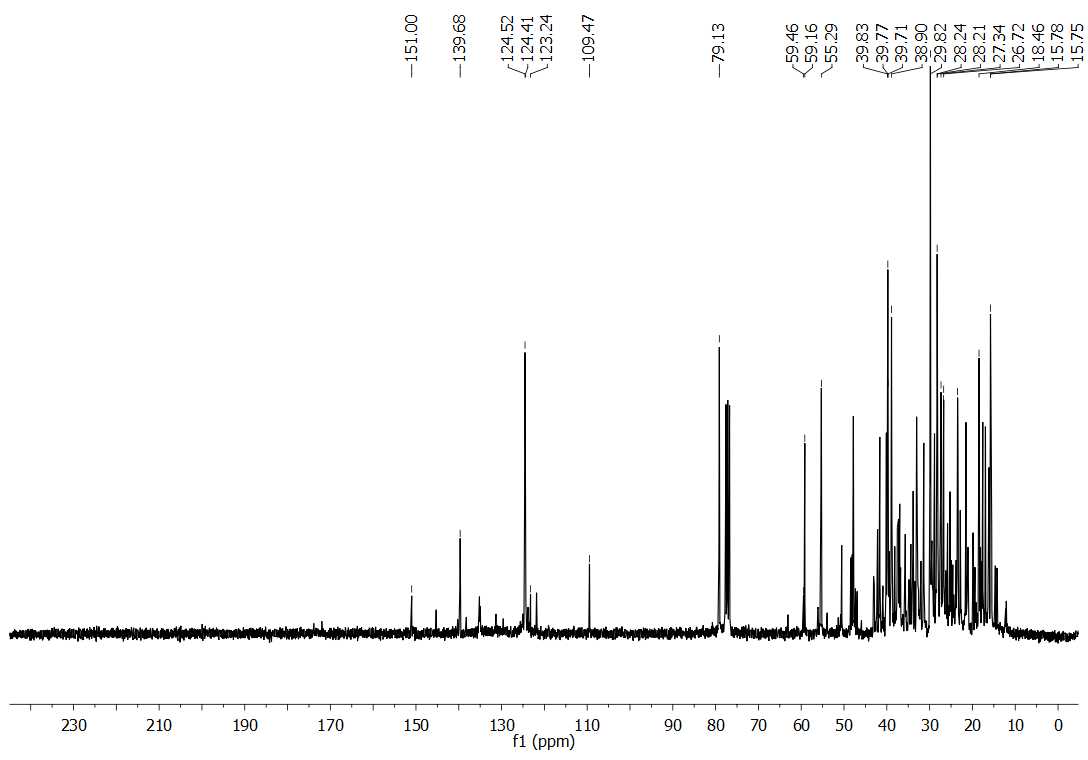


**Figure 38S.** HPLC-DAD-MS/MS (positive mode) profiles of ethanol extracts and phases of *M. albicans*, highlighting the presence of pheophorbides **20** and **21**.

Peak numbers refer to compounds listed in Table 4. LEE = Leaves-ethanol extract. LCP= Leaves-chloroform phase. LEP= Leaves-ethyl acetate phase. LHP= Leaves-hydromethanolic phase. SEE = Stems-ethanol extract. SCP= Stems-chloroform phase. SEP= Stems-ethyl acetate phase. SHP= Stems-hydromethanolic phase.


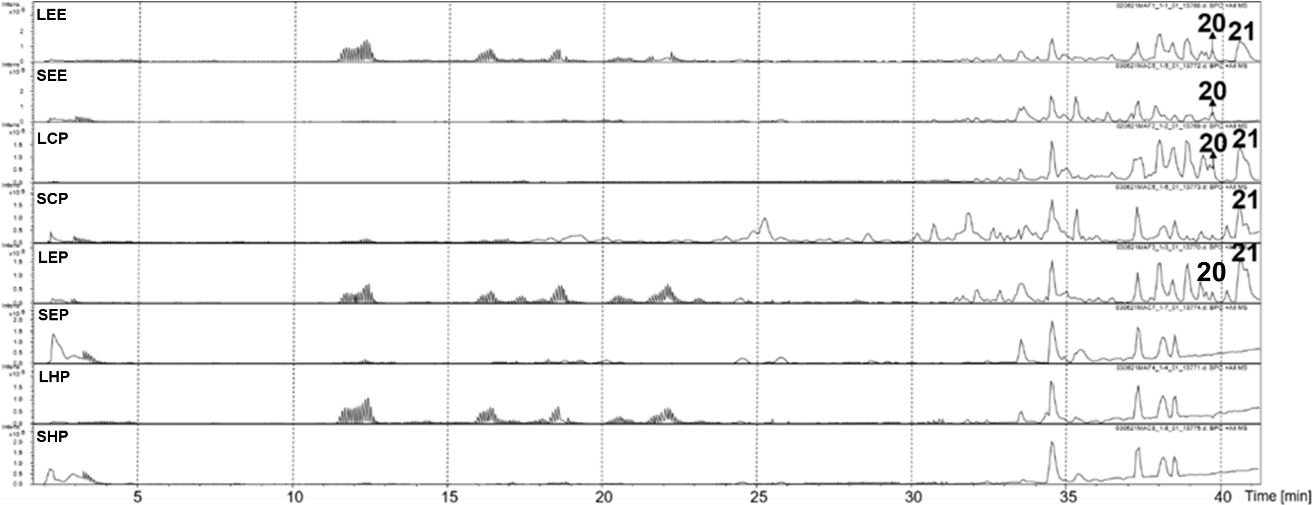

Supplement: Supplementary file 1 — Additional file 1: General experimental procedures. Figure 1S. - Figure 38S. [file 12906_2023_4147_MOESM1_ESM.docx]
